# Immunological Determinants of Oncogenic Virus-Driven Cancers in Africa: Mechanisms, Co-Infections and Public Health Challenges

**DOI:** 10.3390/pathogens15080800

**Published:** 2026-07-28

**Authors:** Victor Ayodele Aliyu, Olalekan Chris Akinsulie, Babatunde Ibrahim Olowu, Ibrahim Idris, Favour Akinfemi Ajibade, Pius I. Babawale, Oluwawemimo Adebowale, Charles Egede Ugwu, Chizaram Blessing Ukauwa, Onyedikachi Emmanuel Itumo, Peter Arinze Oge, Sammuel Shahzad, Chizobam Lilian Chukwu, Toyin Florence Ayandokun, Joy Taiye Aliyu, Peace Kehinde Aliyu, Jesuferanmi Mary Akinsulie, Muhammad Ipoola Adeyemi, Olamilekan Gabriel Banwo

**Affiliations:** 1 School of Molecular Biosciences, College of Veterinary Medicine, Washington State University, Pullman, WA 99164, USA; 2 Department of Veterinary Microbiology and Pathology, College of Veterinary Medicine, Washington State University, Pullman, WA 99164, USA; olalekan.akinsulie@wsu.edu (O.C.A.);; 3 School of Veterinary Medicine, Texas Tech University, Amarillo, TX 79106, USA; 4 Department of Microbiology and Molecular Genetics, The Robert Larner, M.D. College of Medicine, University of Vermont, Burlington, VT 05405, USA; 5 Department of Pathobiological Sciences, School of Veterinary Medicine, Louisiana State University, Barton Rouge, LA 70803, USA; 6 Department of Veterinary Public Health and Preventive Medicine, College of Veterinary Medicine, Federal University of Agriculture Abeokuta, Abeokuta 110124, Ogun State, Nigeria; 7 Paul G. Allen School for Global Health, College of Veterinary Medicine, Washington State University, Pullman, WA 99164, USA; 8 Department of Animal Health and Production, Faculty of Veterinary Medicine, University of Abuja, FCT, Abuja 900102, Nigeria; 9 Department of Public Health, Middlesex University London, London NW4 4BT, UK; 10 Department of Human Anatomy, University of Cross Rivers State, Yala 550107, Cross Rivers State, Nigeria; 11 Department of Medical Microbiology, Imo State University, Owerri 460222, Imo State, Nigeria; 12 Department of Chemistry, College of Arts and Sciences, Washington State University, Pullman, WA 99164, USA; 13 Department of Medicine and Surgery, Faculty of Clinical Sciences, Obafemi Awolowo University, Ile-Ife 220282, Osun State, Nigeria; 14 School of Health Sciences and Society, University of Suffolk, Ipswich IP3 0AQ, UK; 15 Department of Biological Sciences, Faculty of Natural Science and Technology, University of Bergen, 5020 Bergen, Norway; 16 Department of Veterinary Medicine, Faculty of Veterinary Medicine, University of Ibadan, Ibadan 200132, Oyo State, Nigeria

**Keywords:** viral oncogenesis, human papillomavirus (HPV), hepatitis B virus (HBV), Epstein–Barr virus (EBV), sub-Saharan Africa

## Abstract

Oncogenic viruses contribute to approximately 20% of human cancers globally, with their impact falling disproportionately on populations in Sub-Saharan Africa. In this region, cervical cancer, hepatocellular carcinoma, endemic Burkitt lymphoma, and Kaposi sarcoma represent major causes of cancer-related morbidity and mortality, driven by persistent infection with human papillomavirus (HPV), hepatitis B and C viruses (HBV/HCV), Epstein–Barr virus (EBV), Kaposi sarcoma-associated herpesvirus (KSHV), and human T-lymphotropic virus-1 (HTLV-1). This review synthesizes current insights into the immunological mechanisms that underpin viral carcinogenesis in Africa, emphasizing how defective viral clearance, chronic immune activation, and immune evasion arise from the convergence of region-specific co-infections, host genetic diversity, and environmental exposures. We examine the mechanistic roles of HIV-associated CD4^+^ T cell depletion, malaria-induced perturbation of antiviral T cell immunity, helminth-driven T helper 2 polarization, and tuberculosis-associated inflammatory signaling in promoting viral persistence and malignant transformation. In addition, the influence of the extensive diversity of African human leukocyte antigens (HLA) and cytokine gene polymorphisms on antiviral immune responses and cancer susceptibility was discussed. We also assessed how virus-associated tumors establish profoundly immunosuppressive microenvironments characterized by impaired antigen presentation and the dominance of immune checkpoint pathways. Finally, we examined how gaps in vaccination, screening, and diagnostic capacity intersect with immunological vulnerability across Africa, contributing to the burden of infection-associated cancers. These challenges position Africa as a critical setting for developing targeted, genotype-inclusive public health interventions and reducing global cancer disparities through advances in immunoprevention and immunotherapy.

## 1. Introduction to the Global Landscape of Viral Carcinogenesis and Disproportionate Cancer Burden in Africa

**Figure 1:**
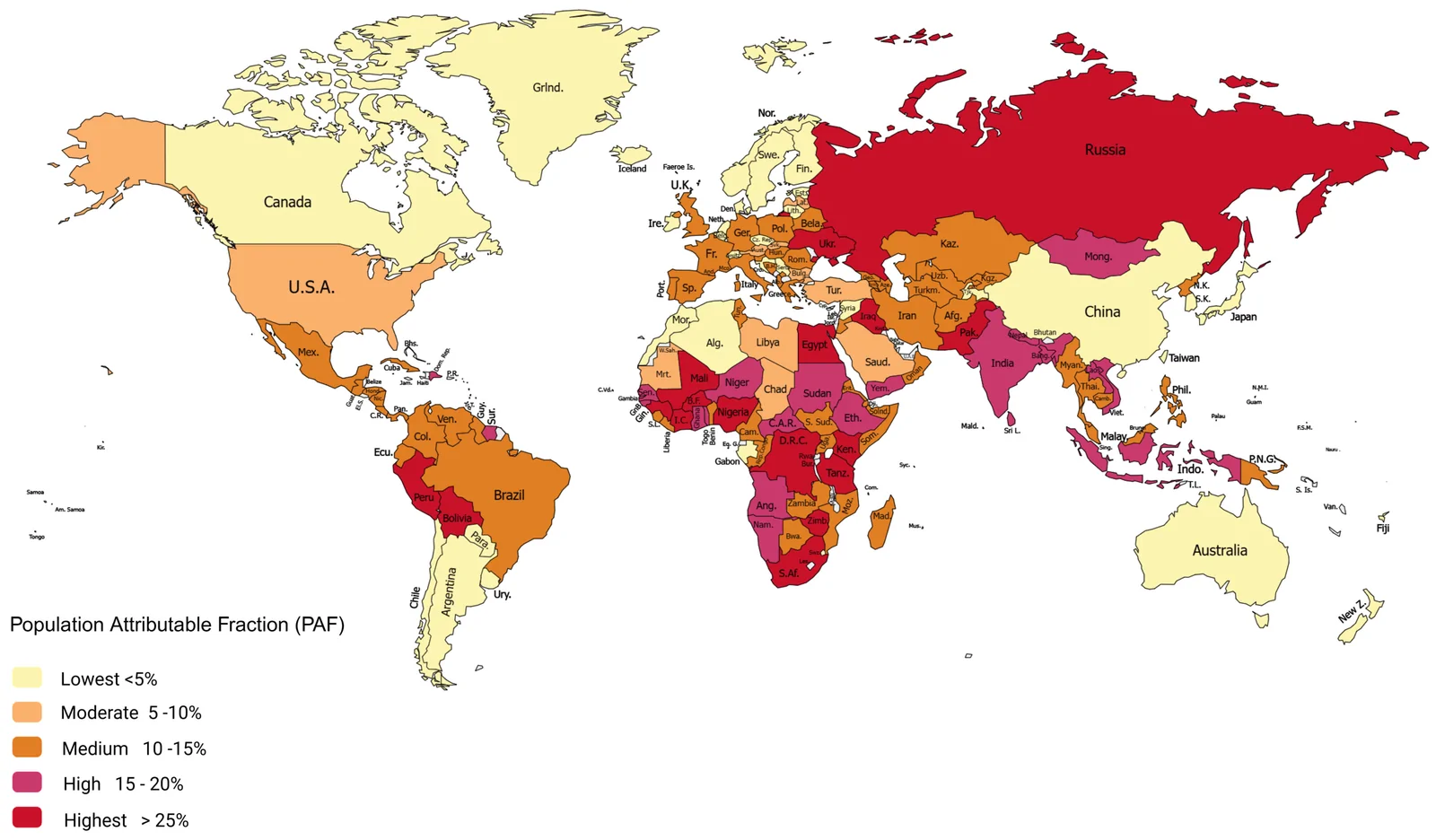
Global burden of virus-associated cancers and the disproportionate impact on Africa. The choropleth map depicts country-level population attributable fractions (PAFs) for cancers associated with infectious agents, grouped into five burden categories ranging from low (<5%) to very high (>25%). The highest burdens are concentrated across sub-Saharan Africa, with additional hotspots in parts of South America and Asia. This distribution underscores the substantial contribution of infection-driven malignancies to the cancer burden in Africa and provides the epidemiological context for examining how oncogenic viruses, co-infections, host immunogenetic diversity, and immune dysregulation contribute to viral persistence and cancer development.

Globally, viral-associated malignant cancer represents a significant burden of cancer worldwide, representing about 20% with a higher proportion in developing nations [1,2]. These groups of viruses are referred to as oncogenic viruses, which include human papillomavirus (HPV), hepatitis B virus (HBV), hepatitis C virus (HCV), Epstein–Barr virus (EBV), Kaposi sarcoma-associated herpesvirus (KSHV), and human T-lymphotropic virus type-1 (HTLV-1) [2]. Despite the global prevalence of these viruses, their malignant effects are disproportionately spread, with sub-Saharan Africa facing a greater rate of virus-associated malignancies [3]. This geographic disparity is evident at the global level, where the population-attributable fraction of infection-related cancers is substantially higher across many regions of Africa compared with most high-income regions (Figure 1). This pattern reflects the intersection of endemic viral exposure, co-infection burden, host immunogenetic diversity, and persistent limitations in cancer prevention and control programs.

Viral carcinogenesis results from complex interactions between persistent viral infection and host immune responses. An essential component in malignant transformation is the deficiency of immune surveillance to eradicate infected or transformed cells, facilitating the persistence of chronic infection [4]. These viruses adopt many immune evasion tactics, such as downregulating antigen presentation pathways, limiting interferon signaling, activating regulatory cytokines, and generating latency [5]. Furthermore, chronic infection induces inflammation, which serves a dual function in cancer development. It can promote tumor growth by establishing an environment abundant in cytokines and growth factors that facilitate cancer cell survival and proliferation, while simultaneously activating antitumor immune responses, a process utilized in cancer immunotherapy [6].

The burden of these malignancies is particularly evident in Africa. Cervical cancer, caused by chronic infection with highly susceptible HPV genotypes, continues to be the largest contributor to cancer mortality in women in sub-Saharan Africa [7]. Chronic HPV infection demonstrates poor cell-mediated immune responses, specifically weakened CD4^+^ T-helper and CD8^+^ cytotoxic T-cell functions necessary for viral destruction [8]. Inadequate access to vaccination, screening, and treatment contributes to late-stage diagnoses [9]. Furthermore, another major virus-associated cancer with significant burden in Africa is the hepatocellular carcinoma and is attributed to chronic infection with HBV or HCV, consumption of aflatoxin-contaminated foods, excessive alcohol intake, obesity, type 2 diabetes, and smoking [10]. Endemic Burkitt lymphoma represents a distinct African model of infection-induced oncogenesis. Although EBV infection is almost ubiquitous globally, its oncogenic expression as endemic Burkitt lymphoma is predominantly found in equatorial Africa [11]. Inadequate transfer of maternal antibodies against EBV and indications of elevated viral reactivation occur when certain mothers are infected with malaria during pregnancy, potentially leading to earlier onset and elevated viral load infections in infancy. Acute, uncomplicated malaria has been linked to the lytic reactivation of EBV and an increased incidence of detectable viremia episodes [12].

The disproportionate burden of virus-associated cancers observed across Africa cannot be explained solely by differences in viral prevalence. Rather, it reflects the convergence of persistent oncogenic viral infections with a uniquely complex immunological landscape shaped by endemic co-infections, extensive host genetic diversity, early-life pathogen exposure, and structural inequities in healthcare access (Figure 1). In many African settings, chronic exposure to HIV, malaria, helminths, and tuberculosis generates overlapping networks of immune activation, immune suppression, and immune polarization that can profoundly influence viral persistence, immune surveillance, and malignant transformation. Simultaneously, exceptional diversity within HLA loci and immune regulatory genes may further modulate susceptibility to infection, cancer progression, and therapeutic responsiveness. Despite these interactions, the immunological mechanisms linking viral infection to cancer development remain incompletely understood and are frequently extrapolated from studies conducted outside Africa. This review synthesizes current evidence on the immunological determinants of oncogenic virus-driven cancers in Africa, focusing on the interplay between viral immune evasion, co-infection-mediated immune modulation, host immunogenetic variation, and the tumor immune microenvironment. By examining these interconnected processes, we highlight Africa not only as a region disproportionately affected by virus-associated malignancies, but also as a uniquely informative setting for understanding the fundamental immunology of viral carcinogenesis and for developing globally relevant prevention, diagnostic, and therapeutic strategies.

## 2. Major Oncogenic Viruses Driving Cancer Burden in Africa

### 2.1. Human Papillomavirus (HPV)

Human papillomavirus (HPV) is a small, non-enveloped, double-stranded DNA virus of the *Papillomaviridae* family with a marked tropism for cutaneous and mucosal epithelial cells. Persistent infection with high-risk HPV (hrHPV) genotypes can drive malignant transformation [13]. More than 200 HPV genotypes have been identified and are broadly classified as low-risk (lrHPV) or high-risk (hrHPV) according to oncogenic potential. While lrHPV types, particularly HPV6 and HPV11, are primarily associated with benign lesions, hrHPV genotypes including HPV16, HPV18, HPV31, HPV33, HPV35, HPV45, HPV52, and HPV58 are implicated in cervical and other anogenital and oropharyngeal cancers [14]. HPV is the established etiological agent of cervical cancer and contributes substantially to the global burden of anal, vulvar, vaginal, penile, and oropharyngeal malignancies [14].

Sub-Saharan Africa (SSA) bears one of the highest cervical cancer burdens worldwide, with nineteen of the twenty countries reporting the highest incidence located on the continent [15,16,17]. SSA records more than 75,000 new cervical cancer cases and over 50,000 deaths annually, with the highest incidence rates observed in Southern and Eastern Africa [18,19]. Overall, approximately 15.8% of all cancers diagnosed in SSA are attributable to HPV infection, underscoring the disproportionate oncologic burden imposed by this virus in the region [20,21,22].

Although HPV16 and HPV18 account for approximately 70% of cervical cancers globally, African populations display distinct genotype distributions. A meta-analysis involving over 25,000 women from 23 African countries demonstrated increasing prevalence of HPV16 and HPV18 from women with normal cytology to those with invasive cervical cancer [14]. However, unlike many non-African populations, additional hrHPV genotypes, particularly HPV35, HPV52, and HPV58, contribute substantially to the African disease burden and collectively account for approximately 4–10% of invasive cervical cancers, despite incomplete representation in earlier vaccine formulations [18]. Marked geographic heterogeneity exists, with HPV prevalence among women with normal cytology ranging from 3.2% in Sudan to 47.9% in Guinea [14,23], while hrHPV prevalence in West Africa ranges from approximately 10.7% in Ghana to over 80% in selected high-risk populations in Nigeria and Burkina Faso [24,25]. A multicountry study from Ghana, Nigeria, and South Africa reported HPV positivity in 93.7% of invasive cervical cancers, with HPV16 (51.2%) and HPV18 (15.0%) remaining the predominant genotypes [25].

HIV coinfection substantially amplifies HPV persistence, alters genotype distribution, and accelerates disease progression across Africa. In Kenya, pooled hrHPV prevalence among women living with HIV reached 64% [26], while among female sex workers in Mombasa, hrHPV prevalence was significantly higher in HIV-positive than HIV-negative women (73.3% versus 45.5%), with HPV16/18 prevalence more than doubled among women living with HIV [27]. Similar observations have been reported in Tanzania and South Africa, where more than half of HIV-positive women harbor at least one hrHPV genotype [28]. In Mali, hrHPV prevalence reached 63%, with HPV35, HPV31, and HPV51/52/56 occurring more frequently among women living with HIV than among HIV-negative women [29]. These findings highlight the synergistic interaction between HIV-associated immunosuppression and persistent hrHPV infection in accelerating cervical carcinogenesis in African populations.

Prophylactic vaccination remains the most effective strategy for preventing HPV-associated malignancies. Currently licensed vaccines include the bivalent Cervarix^®^ (HPV16/18), quadrivalent Gardasil^®^ (HPV6/11/16/18), and nonavalent Gardasil 9^®^, which additionally targets HPV31, HPV33, HPV45, HPV52, and HPV58 [30,31]. These vaccines significantly reduce persistent infection, high-grade cervical lesions, anogenital warts, and multiple HPV-associated cancers [32,33]. However, vaccine implementation across Africa remains heterogeneous because of disparities in healthcare infrastructure, financing, and access, despite substantial support from the World Health Organization and Gavi [34].

At the molecular level, persistent hrHPV infection frequently results in integration of viral DNA into the host genome, leading to sustained expression of the E6 and E7 oncoproteins. These proteins inactivate the tumor suppressors p53 and retinoblastoma protein (pRb), disrupt cell-cycle regulation, inhibit apoptosis, and promote genomic instability [35,36]. HPV also establishes a localized immunosuppressive microenvironment by downregulating antigen presentation pathways and altering cytokine networks, processes that may be further exacerbated by vaginal microbiome dysbiosis, chronic mucosal inflammation, and HIV coinfection [37]. Additionally, E6 and E7 promote IL-23 production and suppress cytotoxic T-cell responses, facilitating viral persistence and malignant progression [38].

### 2.2. Hepatitis B and C Viruses (HBV/HCV)

Hepatitis B virus (HBV) and hepatitis C virus (HCV) are hepatotropic viruses that infect hepatocytes and are the principal viral causes of chronic hepatitis, cirrhosis, and hepatocellular carcinoma (HCC), a leading cause of cancer-related mortality across Africa [39]. HBV is an enveloped, partially double-stranded DNA virus that replicates through reverse transcription of an RNA intermediate, whereas HCV is an enveloped, positive-sense single-stranded RNA virus of the *Flaviviridae* family. Together, these viruses account for most primary liver cancers worldwide, with their oncogenic potential in Africa further amplified by aflatoxin exposure, alcohol use, metabolic disease, HIV coinfection, and chronic immune activation [40].

The epidemiology of chronic viral hepatitis in Africa is highly heterogeneous. Several countries, including Chad, Liberia, South Sudan, Togo, Mauritania, and Guinea, remain hyperendemic for HBV, with hepatitis B surface antigen (HBsAg) prevalence exceeding 8% [41]. A meta-analysis among African blood donors reported the highest pooled HBsAg prevalence in West Africa (10.1%), followed by Central (7.8%) and East Africa (4.9%), with lower estimates in Southern (2.5%) and North Africa (1.7%) [42]. Within East Africa, HBV prevalence averages approximately 6%, with Kenya and Uganda reporting rates as high as 8.5% [43]. Among West African children aged 0–16 years, pooled HBV prevalence is estimated at 5%, reaching 10% in Benin and 7% in Nigeria, while prevalence among HIV-coinfected children approaches 9% [44]. In sub-Saharan Africa, most chronic HBV infections are acquired during infancy or early childhood through vertical or horizontal transmission, resulting in prolonged viral persistence and increased lifetime risk of cirrhosis and HCC [45]. Disease progression is further exacerbated by hepatitis D virus (HDV) coinfection, particularly in Central Africa, where HDV seroprevalence among HBsAg-positive individuals reaches 25.6% in the general population and 37.8% among patients with liver disease [46].

HCV epidemiology in Africa is similarly heterogeneous. Egypt historically carried the world’s highest HCV burden, with adult seroprevalence reaching 14.7% before implementation of national elimination programs [47,48]. Following nationwide screening and large-scale direct-acting antiviral (DAA) treatment campaigns, active infection prevalence declined by approximately 93% to 0.38%, making Egypt the first country to achieve the World Health Organization’s “gold-tier” status toward HCV elimination [49,50]. Outside Egypt, HCV prevalence remains highest in parts of Central and West Africa, including Cameroon, Gabon, Angola, Burkina Faso, and Benin [51]. A recent meta-analysis estimated an overall HCV seroprevalence of 2.3% across sub-Saharan Africa, with regional estimates ranging from 2.9% in Western Africa and 2.7% in Eastern Africa to 0.8% in Southern Africa [52]. In Africa, HCV infection disproportionately affects older adults, with prevalence reaching 16.2% among individuals older than 65 years, and is more common in rural populations (6.6%) than urban populations (2.9%) [52].

Effective preventive and therapeutic interventions exist for both infections, although access remains uneven across the continent. Universal HBV vaccination, particularly timely administration of the hepatitis B birth dose vaccine, substantially reduces chronic infection, yet coverage remains inconsistent because of gaps in healthcare infrastructure, maternal screening, and vaccine delivery systems [53,54]. For chronic HBV infection, nucleos(t)ide analogs such as tenofovir and entecavir effectively suppress viral replication and reduce progression to cirrhosis and HCC. Similarly, DAAs cure more than 95% of HCV infections, although limited diagnostic capacity, high treatment costs, and inadequate screening continue to constrain their impact in many African settings [55].

At the molecular level, HBV acts as both a direct and indirect carcinogen. Integration of HBV DNA into the host genome can induce insertional mutagenesis, chromosomal instability, and dysregulation of host oncogenes, while persistent infection drives chronic necroinflammatory liver injury [56]. In contrast, HCV does not integrate into the host genome but promotes carcinogenesis through sustained inflammation, oxidative stress, fibrosis, and disruption of cellular signaling pathways [57,58]. Both viruses evade innate and adaptive immune responses by impairing antigen presentation, disrupting interferon signaling, and promoting T-cell exhaustion, thereby facilitating lifelong persistence and increasing the risk of malignant transformation [59,60]. These immune evasion mechanisms are often intensified by HIV coinfection and other comorbidities common in Africa, contributing to the continent’s disproportionately high burden of HBV- and HCV-associated HCC [40,59].

### 2.3. Epstein–Barr Virus (EBV)

Epstein–Barr virus (EBV), formally designated Human Gammaherpesvirus 4, is an enveloped, double-stranded DNA virus belonging to the *Herpesviridae* family that establishes lifelong latent infection in more than 90% of the global population [61,62]. EBV exhibits tropism primarily for B lymphocytes and epithelial cells, where it persists within the memory B-cell compartment. Although primary infection is often asymptomatic, persistent latent infection is associated with several malignancies, including Burkitt lymphoma (BL), Hodgkin lymphoma (HL), nasopharyngeal carcinoma (NPC), gastric carcinoma, and a subset of post-transplant lymphoproliferative disorders [63]. In Africa, EBV assumes particular public health significance because of its central role in endemic Burkitt lymphoma (eBL), the most common childhood cancer in many regions of equatorial sub-Saharan Africa [64].

EBV infection is nearly universal across African populations, with most children seroconverting within the first three years of life, substantially earlier than in many high-income settings where primary infection is often delayed until adolescence [65]. This early age of infection, coupled with intense exposure to co-endemic pathogens, creates a unique epidemiological landscape for EBV-associated malignancies on the continent. Within the equatorial “Burkitt lymphoma belt,” which extends across regions of Uganda, Kenya, Tanzania, Malawi, and parts of Central Africa, BL accounts for up to 50% of childhood cancers, and approximately 90–95% of cases are EBV-positive [3,66,67,68]. Annual incidence rates of eBL in these highly endemic regions reach 4–5 cases per 100,000 children under 18 years of age, with peak incidence occurring between 4 and 7 years of age [3].

A defining feature of African EBV epidemiology is its close interaction with *Plasmodium falciparum* malaria. Repeated exposure to holoendemic malaria impairs EBV-specific immune surveillance, resulting in elevated viral loads and prolonged expansion of latently infected B cells [69]. Chronic malaria infection induces polyclonal B-cell activation and dysregulates activation-induced cytidine deaminase (AID), thereby increasing the likelihood of MYC/Ig chromosomal translocations that are characteristic of eBL [69]. Population-based studies from northern Uganda, where eBL is highly endemic, have documented asymptomatic *P. falciparum* prevalence approaching 55% among children younger than 15 years, illustrating the intensity of malaria exposure in regions where EBV-associated lymphomagenesis occurs [70]. Furthermore, a case–control study in Blantyre, Malawi demonstrated that increasing antibody titers against both EBV and malaria were significantly associated with higher odds of Burkitt lymphoma, while HIV-positive children exhibited markedly increased disease risk (Odds Ratio = 12.4) [71]. Comparative analyses from Uganda, Tanzania, and Kenya further suggest that chronic asymptomatic parasitemia, rather than acute clinical malaria, may represent the most relevant malaria exposure contributing to eBL pathogenesis [72].

Beyond eBL, EBV contributes substantially to the burden of other malignancies in Africa. Of the 83,087 Hodgkin lymphoma cases reported globally in 2020, approximately 13% occurred in Africa, accounting for nearly one-fifth of worldwide HL-related deaths [3]. In Rwanda, EBV-encoded RNA in situ hybridization detected EBV in approximately 54% of Hodgkin lymphoma cases, although prevalence varied by histological subtype [73]. EBV-associated NPC and other lymphomas also occur across the continent, particularly in populations affected by HIV-associated immunosuppression, which compromises EBV-specific immune surveillance and facilitates viral persistence [3].

At the molecular level, EBV employs multiple immune evasion strategies to establish persistence and promote malignant transformation. EBV-infected cells downregulate HLA class I expression, reducing recognition by cytotoxic CD8^+^ T cells [74]. Although loss of HLA class I would ordinarily trigger natural killer (NK) cell-mediated killing through “missing-self” recognition, this response may be modified by population-specific KIR-HLA interactions, discussed in later sections of this review [74]. Together with chronic immune activation induced by malaria and HIV coinfection, these immune evasion mechanisms create a permissive environment for EBV-driven oncogenesis in African populations. Despite the substantial burden of EBV-associated malignancies, no licensed prophylactic or therapeutic EBV vaccine currently exists. Consequently, prevention of EBV-associated cancers in Africa currently relies largely on early diagnosis, effective HIV control, and interventions that reduce malaria transmission and exposure.

### 2.4. Kaposi Sarcoma-Associated Herpesvirus (KSHV)

Kaposi sarcoma-associated herpesvirus (KSHV), also known as Human Gammaherpesvirus 8 (HHV-8), is a large enveloped double-stranded DNA gammaherpesvirus and the definitive etiological agent of Kaposi sarcoma (KS), primary effusion lymphoma (PEL), and multicentric Castleman disease (MCD) [75,76]. Following primary infection, KSHV establishes lifelong latency predominantly within endothelial cells and B lymphocytes, where restricted viral gene expression promotes cellular survival, angiogenesis, and immune evasion. Latency-associated proteins, viral microRNAs, viral interferon regulatory factors (vIRFs), and the viral cytokine homolog vIL-6 collectively disrupt host interferon signaling, impair antigen presentation, and establish a highly immunosuppressive and pro-angiogenic microenvironment that favors malignant transformation [77].

The epidemiology of KSHV exhibits marked geographic heterogeneity, with sub-Saharan Africa representing one of the most endemic regions globally. Whereas KSHV seroprevalence is generally below 10% in North America and Europe, adult seroprevalence across African populations ranges from approximately 13% to over 90%, depending on geographic region and population studied [3,76,78]. Lower seroprevalence rates have been reported in parts of West Africa, including 14.3% among pregnant women in Senegal and 12.7% in Burkina Faso [76,79,80]. In contrast, substantially higher rates occur in East and Central Africa, regions encompassed by the historic “Kaposi sarcoma belt” [78]. In Uganda, KSHV seroprevalence ranges from 40% to 50% in the general population and approaches 49% among adults in rural communities, with infection commonly acquired during childhood [76,81]. Similarly, seroprevalence estimates of 46.3% have been reported in rural Tanzania, while rates ranging from 43% to 68% have been documented in Kenya [82,83]. Botswana reports some of the highest prevalence estimates worldwide, with adult seroprevalence ranging from 55% to 90%, whereas lower rates of approximately 21% and 30% have been reported in Mozambique and selected South African populations, respectively [76,84].

The clinical significance of KSHV infection in Africa is closely linked to the HIV epidemic. HIV coinfection is a major determinant of KSHV persistence, reactivation, and disease progression. In Nigeria, KSHV seroprevalence among people living with HIV was estimated at 62%, compared with 26% among HIV-negative individuals [85]. Similarly, KSHV prevalence approaches 90% among HIV-infected populations in Malawi, and seropositivity among patients with Kaposi sarcoma is nearly universal; for example, KSHV antibodies were detected in 100% of Ghanaian KS patients [76]. HIV-mediated CD4^+^ T-cell depletion profoundly impairs immune control of latent KSHV, facilitating viral reactivation, angiogenesis, and tumor development. Consequently, the HIV epidemic drove a dramatic rise in KS incidence across sub-Saharan Africa, where KS remains one of the most common HIV-associated malignancies [86].

Beyond HIV, chronic immune activation resulting from co-endemic infections, including malaria and helminth infections, may further promote KSHV persistence and tumor progression by enhancing systemic inflammation, T-cell exhaustion, and pro-angiogenic signaling pathways [84]. Importantly, restoration of immune function following antiretroviral therapy (ART) frequently results in regression of KS lesions, and expansion of ART programs has substantially reduced KS incidence in several African countries. Nevertheless, delayed HIV diagnosis, incomplete immune reconstitution, and inequitable access to ART continue to sustain a considerable disease burden across the continent [85].

Despite the substantial burden of KSHV-associated malignancies in Africa, no licensed prophylactic or therapeutic vaccine currently exists. Consequently, prevention strategies rely primarily on early HIV diagnosis, prompt initiation of ART, and strengthened cancer surveillance and treatment programs aimed at reducing the incidence and mortality associated with KSHV-driven malignancies.

### 2.5. Human T-Lymphotropic Virus Type 1 (HTLV-1)

Human T-lymphotropic virus type 1 (HTLV-1) is an enveloped, positive-sense single-stranded RNA virus belonging to the *Delta-retrovirus* genus [87]. HTLV-1 displays a highly selective tropism for human CD4+ T-lymphocytes, integrating its proviral DNA into the host genome and establishing a lifelong infection [88]. Unlike many other retroviruses, HTLV-1 propagates primarily through cell-to-cell contact rather than the release of free virions, driving the clonal expansion of infected host cells [89].

HTLV-1 is unevenly distributed globally, with Africa recognized as the largest endemic region worldwide [90,91]. The virus is particularly prevalent in Central, West, and Southern Africa and is estimated to infect between two and five million individuals across the continent [90]. Africa also harbors the greatest HTLV-1 genetic diversity, with six of the seven recognized viral genotypes identified in sub-Saharan Africa and five occurring predominantly in Central Africa [3,92]. The HTLV-1b genotype predominates in countries such as Gabon, Cameroon, the Democratic Republic of the Congo (DRC), and Nigeria, accounting for more than 90% of characterized strains in Gabon and the DRC [91].

The epidemiology of HTLV-1 across Africa is characterized by substantial geographic and demographic heterogeneity. Meta-analyses estimate regional seroprevalence rates of approximately 4.16% in Central Africa, 2.66% in West Africa, and 1.56% in Southern Africa [93,94]. Some of the highest prevalence estimates have been reported in rural Central Africa, particularly in Gabon and the DRC, where population-based studies documented seroprevalence rates ranging from 7% to 25%, especially among older women [91,95,96]. In contrast, lower prevalence rates have generally been reported in urban populations and among pregnant women, highlighting pronounced urban-rural differences in transmission [97]. In West Africa, seropositivity among blood donors and pregnant women typically ranges from 1% to over 5%, although substantially higher estimates have been reported in Guinea-Bissau [80,98,99,100,101]. A systematic review encompassing 25 African countries found HTLV-1 seroprevalence ranging from 0% to 17%, underscoring the marked heterogeneity in transmission patterns across the continent [102]. Interpretation of serological estimates should, however, be undertaken cautiously, as antibody cross-reactivity with malaria antigens may confound prevalence estimates in some endemic settings [103,104,105].

HTLV-1 is the etiological agent of adult T-cell leukemia/lymphoma (ATL), an aggressive malignancy characterized by clonal proliferation of transformed CD4^+^ T cells and poor responsiveness to conventional chemotherapy [106]. Unlike oncogenic viruses that rely on sustained viremia, HTLV-1 persistence is maintained through proviral integration and silent mitotic expansion of infected T-cell clones, thereby minimizing antigen exposure and immune recognition [107]. Viral regulatory proteins, particularly Tax and HBZ, promote malignant transformation by dysregulating NF-κB signaling, enhancing cellular proliferation, and inhibiting apoptosis. In parallel, HTLV-1 impairs antiviral immunity through dendritic-cell dysfunction, chronic inflammatory cytokine production, and disruption of effective T-cell responses [107]. HIV coinfection may further compromise immune surveillance, potentially exacerbating HTLV-1-associated disease progression and ATL risk in endemic African populations [108].

Despite the substantial burden of HTLV-1 infection in Africa, HTLV-1-associated diseases, including ATL and HTLV-1-associated myelopathy/tropical spastic paraparesis (HAM/TSP), remain considerably underdiagnosed across many healthcare settings [90]. No licensed prophylactic vaccine currently exists. Consequently, prevention strategies rely primarily on reducing mother-to-child transmission through breastfeeding interventions, screening blood products, promoting safe sexual practices, and increasing awareness among healthcare providers. Expanded surveillance, improved diagnostic capacity, and strengthened cancer registries are urgently needed to define the true burden of HTLV-1-associated malignancies across the continent.

Collectively, these oncogenic viruses employ diverse mechanisms to establish persistent infection, evade immune surveillance, and promote malignant transformation. Despite differences in viral taxonomy and cellular tropism, several common themes emerge, including the establishment of latency, disruption of antigen presentation pathways, induction of chronic inflammation, and exploitation of host immunoregulatory networks. A comparative summary of the major oncogenic viruses, their immune evasion strategies, associated malignancies, and key oncogenic cofactors in Africa is presented in Table 1.

## 3. Co-Infections and Syndemic Immune Interactions Unique to the African Population

The African continent bears a disproportionately high burden of infectious diseases, creating a complex immunological landscape characterized by frequent and often simultaneous co-infections that profoundly influence host antiviral immunity and cancer susceptibility. In contrast to many high-income settings, where oncogenic viral infections frequently occur in relatively immunologically controlled environments, populations across sub-Saharan Africa are commonly exposed to chronic infections including HIV, malaria, tuberculosis, and helminthiasis alongside persistent oncogenic viral infections. These overlapping infectious pressures generate dynamic cycles of immune activation, immune suppression, and immune polarization that collectively shape viral persistence, immune surveillance, and malignant transformation [114]. Consequently, oncogenic viral pathogenesis in Africa cannot be understood in isolation from the broader infectious and immunological context in which these viruses persist.

### 3.1. HIV as an Immunological Cofactor in Viral Oncogenesis

In Africa, HIV infection is the major immunological cofactor in virus-associated malignancies [112] where the adaptive immune coordination necessary to regulate oncogenic viruses like HPV, EBV, and KSHV is compromised by the progressive depletion of CD4 T-helper cells [86]. Loss of CD4^+^ T cell impairs germinal center formation, antibody maturation, antiviral memory maintenance, and CD8^+^ cytotoxic T lymphocyte (CTL) functionality, thereby weakening immune surveillance against virally infected and transformed cells [115]. Persistent immune activation during chronic HIV infection further accelerates T cell exhaustion and dysregulation of cytokine signaling pathways, even in individuals receiving antiretroviral therapy (ART).

The oncogenic synergy between HIV and KSHV is particularly evident in sub-Saharan Africa, where regions with high HIV prevalence experienced dramatic increases in KS incidence following the HIV epidemic [116]. Population-based cancer registry data from Uganda and Zimbabwe show that KS incidence rose approximately 20-fold within 10–15 years of the HIV epidemic reaching these populations, transforming KS from an uncommon tumor into the most frequently diagnosed cancer in men and the second most frequent in women in these settings [117]. The clinical-epidemiological evidence for this cofactor effect is unusually direct and well-documented. Similarly, in South Africa, KS incidence rose at least three-fold between 1988 and 1996 alone as the HIV epidemic expanded [118]. In Harare, Zimbabwe, KS incidence reached a peak of 50.8 per 100,000 in men and 20.3 per 100,000 in women during the pre-antiretroviral therapy era [119]. Globally, people living with HIV have been estimated to carry approximately a 500-fold higher risk of KS than the general population, and roughly three-quarters of all KS cases worldwide are attributable to HIV co-infection even in the current ART era [120]. HIV-associated immunosuppression facilitates uncontrolled KSHV replication and persistence, while HIV-derived proteins, including the trans-activator of transcription (Tat), directly enhance angiogenesis, inflammatory signaling, and tumor progression [121].

Importantly, the scale-up of ART has been associated with a measurable decline in KS incidence across sub-Saharan Africa, with cancer registry data indicating an average reduction of approximately 27% between the periods 2000–2010 and 2011–2016, coinciding with expanding ART coverage [118]. These observations provide population-level evidence that restoration of CD4^+^ T-cell function reduces KSHV-driven oncogenesis. Nevertheless, residual immune dysfunction and incomplete restoration of antiviral immunity continue to sustain elevated cancer risk among people living with HIV despite ART [113], highlighting the long-term consequences of chronic immune dysregulation in virus-associated malignancies.

### 3.2. Malaria-EBV Interactions and Endemic Burkitt Lymphoma

The interaction between holoendemic *Plasmodium falciparum* malaria and EBV represents one of the clearest examples of co-infection-driven oncogenesis unique to Africa. In malaria-endemic regions, repeated *P. falciparum* exposure induces chronic immune activation and polyclonal B cell expansion [122], thereby increasing the pool of EBV-infected B lymphocytes susceptible to malignant transformation [123]. Simultaneously, malaria impairs EBV-specific CD8^+^ T cell responses that are essential for maintaining latency control, resulting in increased viral replication and expansion of latently infected B cell populations [124].

Persistent immune stimulation further promotes hyperexpression of activation-induced cytidine deaminase (AID) within germinal center B cells. Chronic AID upregulation increases genomic instability and markedly elevates the likelihood of the characteristic t(8;14) chromosomal translocation, which places the *MYC* proto-oncogene under the control of the immunoglobulin heavy-chain locus, leading to constitutive *MYC* expression and malignant transformation [125,126]. Through these combined effects on viral persistence, immune surveillance, and genomic stability, malaria acts not merely as a coincidental co-infection but as a potent immunological amplifier of EBV-driven lymphomagenesis, contributing to the high incidence of endemic Burkitt lymphoma (eBL) in equatorial Africa [127]. Thus, malaria acts not merely as a coincidental co-infection but as a potent immunological amplifier of EBV-driven lymphomagenesis by simultaneously promoting viral persistence and weakening antiviral immune surveillance.

Epidemiological studies strongly support this biological interaction. A case–control study of childhood Burkitt lymphoma in Blantyre, Malawi demonstrated a synergistic effect between EBV and malaria exposure [71]. The odds ratio (OR) for Burkitt lymphoma was 1.4 among children with elevated EBV antibody titers alone and 5.7 among those with elevated malaria antibody titers alone; however, the OR increased to 13.2 among children with elevated antibody responses to both pathogens, indicating a synergistic rather than additive interaction [71]. Importantly, reported use of insecticide-treated mosquito nets was associated with significantly reduced odds of Burkitt lymphoma (OR 0.2), further implicating malaria exposure in eBL pathogenesis [71].

Population-level evidence further reinforces these observations. A systematic review and meta-analysis of Burkitt lymphoma incidence across sub-Saharan Africa found that the large-scale implementation of insecticide-treated bed net (ITN) programs during the 2000s was associated with an approximately 44% reduction in childhood Burkitt lymphoma incidence, with pooled incidence declining from 1.36 to 0.76 cases per 100,000 children following ITN scale-up [128]. Moreover, a Mendelian randomization study conducted in northern Uganda provided genetic evidence supporting a causal relationship between malaria exposure and eBL susceptibility, strengthening the evidence that malaria is a key cofactor in EBV-associated lymphomagenesis [129]. Collectively, these findings suggest that effective malaria control strategies may substantially reduce the burden of EBV-associated Burkitt lymphoma in endemic regions of Africa.

### 3.3. Tuberculosis-Associated Inflammation and Viral Oncogenesis

Tuberculosis (TB) further complicates the immunological landscape of oncogenic viral infections in Africa. Chronic *Mycobacterium tuberculosis* infection is characterized by persistent macrophage activation, granuloma formation, and prolonged production of pro-inflammatory cytokines including TNF-α and IFN-γ [130]. Sustained inflammatory signaling promotes oxidative stress, tissue remodeling, and cellular damage, all of which may enhance carcinogenic processes during chronic viral infection [131,132].

In individuals with chronic HBV or HCV infection, TB-associated inflammation has been proposed as a potential contributor to accelerated hepatic injury and fibrosis. However, direct evidence linking TB-induced inflammation to increased hepatocellular carcinoma (HCC) risk remains limited [133]. The most clearly established interaction between TB and oncogenic viral hepatitis is pharmacological rather than directly carcinogenic. Among patients with chronic HBV infection, anti-tuberculosis therapy is associated with a three- to five-fold increased risk of drug-induced hepatotoxicity compared with TB patients without viral hepatitis, often necessitating treatment interruption or regimen modification [134]. This risk increases substantially in individuals with combined HIV/HCV co-infection, reaching approximately 14-fold higher rates of hepatotoxicity [134].

Given the substantial overlap between TB, HIV, HBV, and HCV endemicity in many African settings, these interactions represent a major clinical challenge. Whether chronic TB-associated inflammation independently accelerates fibrosis progression or HCC development in HBV- or HCV-infected individuals remains insufficiently investigated in African populations and constitutes an important area for future research.

### 3.4. Helminth Infections and Antiviral Immune Polarization

Helminth infections are highly prevalent across many African regions and exert profound immunomodulatory effects that may influence susceptibility to oncogenic viral infections. Chronic helminthiasis promotes strong T helper 2 (TH2)-biased immune responses characterized by elevated production of IL-4, IL-5, IL-10, and TGF-β, alongside expansion of regulatory T cells and alternatively activated macrophages [135]. Although these responses are beneficial in limiting parasite-induced tissue damage, they may simultaneously suppress TH1-mediated antiviral immunity and impair CD8^+^ T cell effector function [136].

Evidence for these interactions is strongest for schistosomiasis. In Africa, *Schistosoma mansoni* coinfection has been associated with more severe liver disease and poorer clinical outcomes among individuals chronically infected with HBV [137]. Similarly, schistosomiasis has been linked to higher HCV viral loads, accelerated liver fibrosis, and increased risk of cirrhosis and hepatocellular carcinoma in HCV-infected individuals [138,139]. By contrast, direct epidemiological evidence linking helminth infections to worse outcomes in HPV- or EBV-associated malignancies remains limited. Nevertheless, the widespread endemicity of helminth infections across Africa suggests that helminth-induced immune modulation may represent an important, but underexplored, contributor to regional differences in virus-associated cancer susceptibility.

Collectively, these co-infections generate a uniquely complex immunological environment in Africa characterized by intersecting pathways of chronic inflammation, immune suppression, and immune exhaustion, which can enhance viral persistence and diminish effective immune surveillance against malignant transformation. These interactions are likely synergistic rather than merely additive, amplifying viral persistence, impairing immune surveillance, and increasing susceptibility to virus-associated malignancies. Consequently, integrated prevention and treatment strategies targeting both oncogenic viruses and endemic co-infections may represent a critical, yet underutilized, approach for reducing cancer burden across the continent.

## 4. Host Genetic and Immunological Diversity in Africans

**Figure 2:**
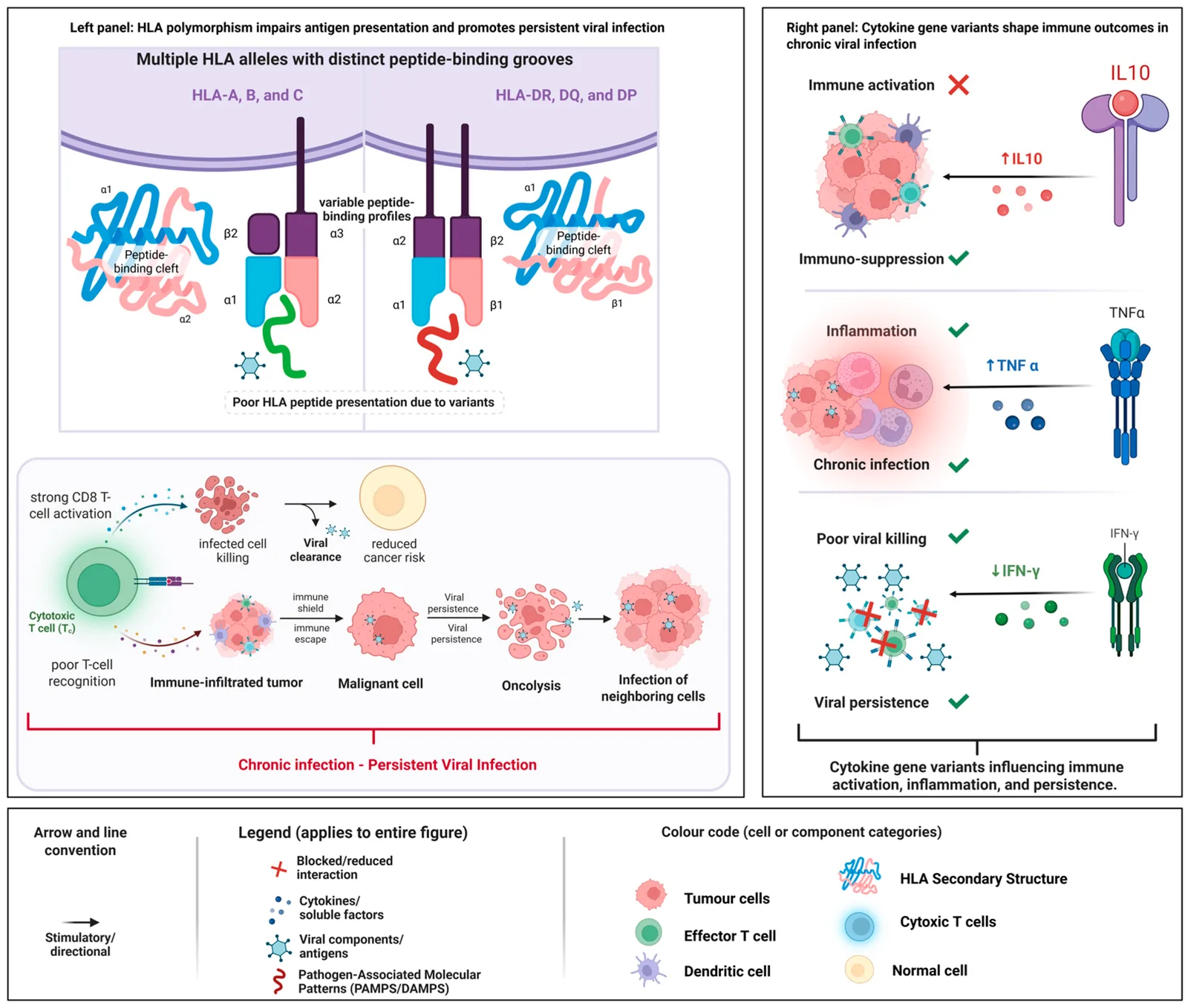
Host genetic diversity modulates antiviral immunity, viral persistence, and cancer susceptibility. (**Left panel**): Polymorphic HLA class I (HLA-A, -B, and -C) and class II (HLA-DR, -DQ, and -DP) alleles exhibit distinct peptide-binding repertoires that influence viral antigen presentation, CD8^+^ T-cell activation, infected-cell killing, and viral clearance. Impaired peptide presentation associated with unfavorable HLA variants may promote immune escape, chronic viral persistence, and malignant transformation. (**Right panel**): Cytokine-associated genetic variation shapes antiviral immune responses and inflammatory outcomes. Increased IL-10 signaling promotes immunosuppression; elevated TNF-α sustains chronic inflammation; reduced IFN-γ signaling compromises antiviral effector function and viral killing, all of these contributing to persistent infection, malignancy and tumor-promoting immune microenvironments.

African populations harbor some of the greatest human genetic diversity globally, with important implications for antiviral immunity and susceptibility to virus-associated cancers [3]. Extensive polymorphism within the human leukocyte antigen (HLA) locus shapes the efficiency of viral peptide presentation, thereby influencing immune recognition, viral clearance, and persistence (Figure 2) [140]. Certain HLA alleles are associated with effective control of oncogenic viruses, whereas others correlate with chronic HBV infection, persistent HPV infection, or impaired EBV-specific cytotoxic T cell responses, thereby increasing cancer susceptibility. In parallel, polymorphisms within cytokine genes, including *IL10*, *TNFα*, and *IFNγ*, influence inflammatory tone, antiviral immunity, and viral persistence.

Beyond susceptibility, host immunogenetic variation also influences the natural history of viral infection, affecting spontaneous viral clearance, disease severity, progression to malignancy, and responses to antiviral or immunotherapeutic interventions. Importantly, the clinical impact of these genetic variants is often modified by factors highly prevalent in African settings, including HIV coinfection, malaria exposure, age at infection, and other endemic comorbidities. Consequently, understanding immunogenetic determinants of viral carcinogenesis in Africa requires consideration of both the continent’s exceptional genetic diversity and its unique epidemiological landscape. This section examines how host genetic and immunological variation shapes viral pathogenesis and contributes to heterogeneity in cancer risk and clinical outcomes across African populations.

### 4.1. HLA Polymorphisms and Viral Control

HLA molecules are central mediators of adaptive antiviral immunity, orchestrating antigen presentation, antibody synthesis, and T cell activation during viral infection [140]. Classical HLA class I molecules (*HLA-A*, *HLA-B*, and *HLA-C*) are ubiquitously expressed on all nucleated cells and consist of two noncovalently associated polypeptide chains: a highly polymorphic α-chain encoded within the major histocompatibility complex (MHC) region on chromosome 6, and a nonpolymorphic β2-microglobulin chain encoded on chromosome 15 [141]. These molecules present endogenous peptides derived from intracellular pathogens, including viruses, to CD8^+^ cytotoxic T lymphocytes (CTLs), thereby facilitating immune recognition and elimination of infected cells [142]. In contrast, classical HLA class II molecules (*HLA-DR*, *HLA-DQ*, and *HLA-DP*) are heterodimeric structures composed of α- and β-chains, both encoded within the MHC locus, and are primarily expressed on professional antigen-presenting cells, including dendritic cells, macrophages, and mature B lymphocytes [143]. HLA class II molecules present peptides generated through endosomal processing of exogenous and intracellular proteins to CD4^+^ T helper lymphocytes, thereby coordinating cellular and humoral immune responses [144]. In addition to immune cells, HLA class II expression has also been observed on epithelial within the pulmonary and gastrointestinal tracts, suggesting broader roles in mucosal immunity and pathogen sensing [145].

The HLA represents the most polymorphic gene family within the human genome [146]. To date, over 30,000 HLA alleles, encoding over 18,000 unique allotypes have been identified [147]. Most nucleotide substitutions found in HLA alleles are concentrated within the exons that code for the peptide-binding groove and the regions that interact with T-cell receptors; notably, the most polymorphic sites are those that influence peptide binding [148]. Structural variability within these domains alters peptide-binding affinity, groove geometry, electrostatic charge distribution, and hydrophobic interactions, thereby determining which viral epitopes can be effectively presented to T lymphocytes. Consequently, individuals carrying different HLA genotypes display marked variability in antigen presentation efficiency, antiviral immune activation, and susceptibility to infectious diseases [141].

African populations harbor the greatest HLA diversity globally, largely reflecting prolonged evolutionary selection imposed by endemic infectious diseases [149]. The immunogenetic heterogeneity has profound implications for susceptibility to oncogenic viral infections and associated malignancies across the continent. Specific HLA alleles and haplotypes have been linked to differential control of HBV, HPV, EBV, KSHV, and HTLV-1 infections, influencing viral persistence, immune escape, and progression toward malignancy [150].

Among HLA class II molecules, HLA-DQ is of particular interest because of its critical role in antiviral CD4^+^ T cell priming. Functional HLA-DQ molecules are heterodimers composed of α- and β-subunits encoded by *HLA-DQA1* and *HLA-DQB1*, respectively [151]. These genes exhibit extensive polymorphism, particularly within exon 2, which encodes the antigen-binding domain [151]. Variants within the HLA-DQ locus have been strongly associated with chronic hepatitis B susceptibility. Genome-wide association studies identified polymorphisms including rs2856718 and rs7453920 as important determinants of chronic HBV persistence, with evidence suggesting that these variants alter HLA-DQ transcriptional activity and antigen presentation efficiency [148,152].

Similarly, persistent high-risk HPV infection has been linked to specific HLA alleles associated with impaired presentation of HPV-derived epitopes to CD8^+^ T cells, thereby facilitating immune evasion and progression toward cervical carcinogenesis [153]. In EBV-associated malignancies, defective EBV-specific CTL responses linked to certain HLA backgrounds may compromise control of latent infection [154], particularly in malaria-endemic regions where chronic immune activation further perturbs antiviral immunity. These interactions underscore how host HLA diversity and environmental infectious pressures may synergistically shape virus-associated cancer susceptibility across African populations.

Beyond viral control, HLA polymorphisms also influence responsiveness to vaccination and immunotherapy. Twin studies conducted in The Gambia demonstrated that both HLA and non-HLA loci contribute substantially to vaccine responsiveness during early life, while environmental exposures influence long-term antibody persistence, particularly against tetanus toxoid [155,156]. Additionally, studies across diverse populations indicate that vaccine-induced antibody responses exhibit significant heritability, although the magnitude of genetic contribution varies between vaccines, highlighting the importance of host immunogenetic variation in shaping adaptive immunity [157,158]. HLA class II molecules play a central role in vaccine responsiveness by presenting vaccine-derived epitopes to CD4^+^ T cells, thereby driving TH1 and TH2 differentiation, B cell activation, plasma cell formation, and immunological memory generation.

Considerable evidence links HLA class II polymorphisms, particularly within the *HLA-DRB1* locus, to variability in responsiveness to hepatitis B and measles vaccination [159]. Approximately 10% of individuals fail to generate protective antibody titers following hepatitis B vaccination, with several studies implicating specific HLA alleles in both primary vaccine responsiveness and revaccination outcomes. Similarly, measles vaccine responsiveness exhibits substantial heritability, including evidence of HLA-associated heterozygote advantage, a phenomenon also observed in HIV and HBV infections [159]. Beyond HLA genes, innate immune pathways, including Toll-like receptor–associated signaling networks, are increasingly recognized as important determinants of vaccine efficacy and potential targets for next-generation vaccine adjuvant development [160,161].

Collectively, HLA polymorphisms constitute major immunological determinants of antiviral immunity, viral persistence, vaccine responsiveness, and susceptibility to virus-associated malignancies in Africa. The remarkable HLA diversity observed across African populations provides both a challenge and an opportunity for understanding viral pathogenesis, improving vaccine design, and advancing precision immunological approaches to cancer prevention and immunotherapy.

#### 4.1.1. High HLA Variability and Differential Viral Clearance

HLA molecules are critical determinants of the cellular immune response to oncogenic pathogens, including human papillomavirus (HPV) and hepatitis B virus (HBV) [141,162]. Genome-wide association studies (GWAS) have demonstrated robust associations between cervical cancer and the 6p21.3 locus harboring the HLA gene cluster [163], emphasizing the central role of antigen presentation in HPV immune surveillance. Because HLA polymorphisms directly influence peptide-binding affinity and epitope presentation to cytotoxic T lymphocytes (CTLs) and natural killer T cells, variation within HLA loci can determine whether viral infections undergo effective immune-mediated clearance or progress toward persistence, immune escape, and malignant transformation [162,163].

HBV provides one of the clearest examples of HLA-mediated viral control. HBV is classified into at least ten genotypes (A–J) based on intergenotypic sequence divergence exceeding 7.5% across the viral genome [164]. Owing to extensive polymorphism within HLA peptide-binding domains, different HLA alleles exhibit variable capacities to bind and present HBV-derived epitopes, thereby influencing antiviral CTL responses and clinical outcomes [165,166]. Advances in next-generation sequencing (NGS) have enabled increasingly precise characterization of HLA diversity and its association with susceptibility to chronic viral hepatitis [167], enabling more precise delineation of allele-specific effects on antiviral immunity. In sub-Saharan Africa, alleles including HLA-A*23:01 (31.4%), HLA-C*07:01 (29.3%), and HLA-C*04:01 (28.6%) are among the most prevalent, as documented in Cameroonian cohorts [168,169,170,171]. Importantly, HLA-A*30:01 and HLA-C*17:01 were significantly enriched in HBV- and HCV-infected individuals in Cameroon, whereas HLA-C*03:04 was markedly more frequent among uninfected controls, implicating this allele as potentially protective against hepatitis virus acquisition, the first such report for HBV and HCV in a West/Central African population [62]. Additional studies have implicated HLA-A*02, HLA-A*03, and HLA-A*31 with outcomes of viral hepatitis [170], consistent with the established predominant role of the HLA-A locus in orchestrating host immune responses to DNA viruses [172,173].

In HIV-1 infection, which indirectly potentiates oncogenic virus-associated cancers through profound immunosuppression, HLA-B alleles represent major determinants of viral control. HLA-B*57:01 and HLA-B*57:03 are strongly associated with elite controller status through presentation of highly conserved, immunodominant Gag epitopes that impose severe fitness costs upon CTL-driven mutational escape [174,175]. A large-scale GWAS of elite controllers confirmed that a 1.9-megabase haploblock tagging HLA-B*57:01 in Europeans corresponded to an analogous haploblock tagging HLA-B*57:03 in African American cohorts, with closely mirrored MHC gene expression profiles, underscoring shared mechanisms of viral control across populations [175]. Conversely, HLA-B*35 alleles (Px motif subtype) have been consistently associated with accelerated HIV disease progression and higher viral set-points, with implications for the burden of AIDS-defining malignancies including Kaposi sarcoma and non-Hodgkin lymphoma in African cohorts [176].

EBV, the aetiological co-factor in endemic Burkitt lymphoma (eBL), demonstrates HLA-restricted immunological control mediated by CTL responses to latent membrane proteins and nuclear antigens. A high-resolution HLA sequencing study of 200 eBL cases and 400 controls in northern Uganda found that cases had significantly lower frequencies of HLA-A*02 and HLA-B*58 alleles, while *HLA-DQA1* homozygosity was associated with increased eBL risk, implicating CD4^+^ T-cell-mediated EBV surveillance as a key protective mechanism [177]. A subsequent GWAS further identified HLA-DQA1*04:01 as a risk variant for childhood eBL in East Africa, reinforcing the importance of MHC class II-governed EBV latency control in this setting where malaria-mediated immune perturbation compounds viral burden [178].

#### 4.1.2. HLA Variability with HPV Persistence and HBV Chronicity

Chronic HBV infection is the leading cause of cirrhosis and hepatocellular carcinoma (HCC) globally, and host immunogenetic factors alongside viral and environmental variables are critical modulators of disease chronicity [140,168]. HLA class I and II diversity at the population level has been consistently associated with differential rates of hepatitis virus clearance versus persistence across multiple cohorts [140,179]. HLA class II molecules are central mediators of these effects: the polymorphic *HLA-DQA1* and *HLA-DQB1* genes, whose exon 2 encodes the antigen-binding site, are particularly polymorphic [147]. The intergenic SNP rs2856718 (between *HLA-DQA2* and *HLA-DQB1*) and the intronic SNP rs7453920 (within *HLA-DQB2* intron 1) have been identified by GWAS as significantly associated with chronic HBV infection [147,152]; transcriptomic data indicate that the rs7453920 A allele correlates with elevated HLA-DQ mRNA in circulating monocytes, enhancing antigen presentation capacity [148]. Multiple studies across diverse populations confirm the association between HLA-DQ polymorphisms and susceptibility to persistent chronic hepatitis B viral infection, though reported allele-specific effects have not been uniform, reflecting population stratification and limited individual study sample sizes [145,146,147,152].

In the context of HPV and cervical cancer, a study of African women characterized the distribution of HLA alleles and their association with prevalent and persistent cervical high-risk HPV (hrHPV) infection [180]. DQA1*01:02 (allele frequency 39%; population frequency 63% in the study cohort) and DQA1*02:01 were associated with increased odds of prevalent hrHPV infection, and both alleles are notably prevalent across sub-Saharan Africa, including in Gabon (50%), Congo (42%), Cameroon (38%), and Kenya (32%) [180]. Conversely, DQA1*05:01 was associated with a 55% reduction in prevalent hrHPV infection odds, though hrHPV demonstrated greater persistence among carriers of this allele, suggesting a potential role in immune evasion and cervical malignancy [180]. DQB1*05:01 was associated with a 66% reduction in persistent hrHPV odds, though this association was attenuated in fully adjusted models [180]. These data indicate that specific HLA alleles modulate viral clearance versus persistence through differential capacity of class I and class II molecules to present HPV peptides to CD8^+^ CTLs and CD4^+^ T helper cells, respectively; downregulation of HLA class I expression, as observed in cervical cancer, enables HPV-infected cells to evade CTL surveillance [180]. Future larger studies characterizing type-specific hrHPV persistence in relation to HLA allelic variants will be essential for informing personalized immunological approaches to cervical cancer prevention [180].

Parallel evidence exists for HLA-driven modulation of HBV outcomes in South African and other African cohorts, where the interplay between HBV genotype diversity and host HLA further complicates the extrapolation of findings from Asian GWAS [164,168]. Although GWAS studies in predominantly Asian populations have identified HLA-DP locus variants as the strongest common genetic determinants of HBV chronicity [158], the higher HLA allelic richness and distinct linkage disequilibrium patterns of African populations well-documented in Cameroon, Harare, Guinea-Bissau, and other cohorts [169,170,171] necessitate Africa-specific GWAS to resolve population-relevant associations. Twin studies from The Gambia further confirm that both HLA and non-HLA loci contribute to vaccine immune response variation in early life [155], with associations at the *HLA-DRB1* locus documented for hepatitis B vaccine response and measles vaccine response, with evidence of heterozygote advantage for measles antibody titers [158].

These findings collectively support the concept that HLA polymorphisms critically shape antiviral immune surveillance and determine whether oncogenic viral infections are efficiently cleared or progress toward chronic persistence. Given that chronic inflammation and prolonged viral replication are major drivers of hepatocarcinogenesis, HLA-associated differences in viral control may substantially contribute to regional disparities in HCC burden across Africa.

### 4.2. Cytokine Polymorphisms and Immunological Heterogeneity in Oncogenic Viral Persistence

Cytokines are immunomodulatory mediators, and functional single nucleotide polymorphisms (SNPs) in cytokine gene promoters could alter transcription factor binding and cytokine production levels, with consequent effects on the host immune response to viral infections and cancer susceptibility [181,182,183,184]. Regardless of antiviral therapy, HBV infection outcomes from viral clearance to chronicity and progression to fibrosis and HCC are influenced by host genetic predisposition, including cytokine gene polymorphisms [181]. In African populations, the allelic architecture of cytokine gene polymorphisms reflects evolutionary pressures of endemic infectious disease and the continental genetic diversity that distinguishes African groups from non-African populations.

#### 4.2.1. Cytokine Genetic Variation and Susceptibility to HBV-Associated Hepatocarcinogenesis

Host cytokine polymorphisms are increasingly recognized as important determinants of HBV persistence, hepatic inflammation, fibrosis progression, and hepatocellular carcinoma (HCC) development [181]. A study in Ghanaian patients with HBV infection examined 13 SNPs across *IL-2*, *IL-10*, *IL-18*, *TNF-α*, *IFN-γ*, and *IFN-γR1* genes in relation to plasma HBV DNA levels and FIB-4 scores (a non-invasive hepatic fibrosis biomarker) [181]. Carriers of the *IL2* rs1479920 GG variant allele exhibited significantly lower median HBV DNA levels compared to AA homozygotes, consistent with the immunostimulatory role of IL-2 in enhancing host antiviral responses [185,186] and suggesting a degree of protective immunological effect from this intronic variant [181].

Promoter polymorphisms within *TNF-α* also appear to influence HBV disease outcomes. The rs1799724 C>T (−857C/T) variant was significantly associated with HBV DNA levels, likely through modulation of TNF-α transcription and downstream regulation of HLA class II expression and viral antigen presentation [187,188,189,190]. Likewise, the TNF-α −308A/G (rs1800629) polymorphism was further associated with FIB-4 scores: carriers of the AA genotype demonstrated lower odds of elevated FIB-4 in both univariate and multivariate analyses, suggesting that the −308A allele may confer a more favorable hepatic fibrosis trajectory, though this contrasts with evidence from other populations linking this allele with increased HCC risk [181,191]. These discordant findings highlight the importance of ethnic background in interpreting cytokine gene associations, and the need for larger cohort studies in African populations to resolve these relationships [181].

TNF-α is produced predominantly by macrophages, monocytes, neutrophils, T lymphocytes, and NK cells, and functions as a principal stimulatory agent facilitating the secretion of downstream cytokines and endothelial adhesion molecule expression [191,192,193]. During chronic HBV infection, excessive TNF-α production drives hepatocyte apoptosis and inflammatory fibrogenesis, thereby promoting cirrhosis and hepatocarcinogenesis [181]. Associations between cytokine gene variants and HBV DNA levels, fibrosis biomarkers, and inflammatory activity observed across IL-10, IL-18, TNF-α, IFN-γ, and IFN-γR1 polymorphisms collectively support the concept that host immunogenetic variation shapes the entire continuum of HBV disease. These polymorphisms influence the probability of spontaneous viral clearance, persistence of chronic infection, progression to cirrhosis, and eventual development of HCC. Moreover, several cytokine variants have been associated with differential responses to antiviral therapy, suggesting that immunogenetic profiling may ultimately contribute to individualized prognostication and therapeutic decision-making in HBV-infected populations [181,194].

#### 4.2.2. Cytokine Variants Shaping HPV Persistence and Cervical Carcinogenesis

Cytokine gene polymorphisms also modulate susceptibility to HPV-driven cervical cancer. Among the most extensively studied are promoter polymorphisms within *IL-10*, particularly −1082A/G (rs1800870), −819T/C (rs1800871), and −592C/A (rs1800872), which significantly influence IL-10 transcriptional activity [195,196,197,198]. IL-10 is a principal anti-inflammatory cytokine with antiangiogenic and immunosuppressive properties that may exert both tumor-suppressive and tumor-promoting effects [199,200].

A case–control study by Stanczuk et al. in an African cohort demonstrated a significant association between the *IL-10*−1082A/G polymorphism and increased cervical cancer risk, with carriers of the A/G genotype at exhibiting heightened susceptibility to disease progression [201]. Subsequent studies corroborated this finding: women carrying A/G and A/G+G/G genotypes demonstrated 2.35- and 2.03-fold elevated cervical cancer risk respectively, while the G allele was independently associated with increased cervical cancer susceptibility primarily in cohorts dominated by persistent hrHPV infection [202]. The −1082G allele has also been significantly associated with cervical cancer in Zimbabwean women [201], while haplotype analyses confirmed associations between IL-10−1082/−819 and TNF-308 haplotypes and susceptibility to cervical carcinogenesis in HPV-infected women [200]. These findings suggest that high IL-10–producing genotypes may facilitate persistent HPV infection by suppressing effective antiviral T cell responses and impairing immune-mediated viral clearance.

Polymorphisms within *TNF-α* similarly influence cervical cancer susceptibility through effects on inflammatory signaling and HPV antigen presentation. TNF-α directly facilitates reduction in HPV gene transcripts, induces apoptosis in HPV-infected cells, amplifies the inflammatory response to HPV, and enhances HPV antigen presentation to effector T cells [203,204,205]. SNPs in the *TNF-α* promoter region—rs361525 (−238 G>A), rs1799964 (−1031 T>C), and rs1800629 (−308 G>A)—alter circulating TNF-α levels and thereby modulate immune recognition of HPV-infected cells [206]. In a Nigerian cohort, the TNF-α −1031 T>C polymorphism was significantly associated with an increased risk of cervical cancer among HIV-negative women. In contrast, the TNF-α −308A>G A allele was identified as a significant risk factor among HIV-positive women, suggesting that the oncogenic effects of TNF-α promoter polymorphisms may be influenced by host immune status. These results contrast with studies from South Africa and Zimbabwe reporting no significant association between the −308 G/A polymorphism and cervical cancer risk [207,208], illustrating the context-dependency of these associations across African populations. Diminished TNF-α levels correlate with impaired HPV antigen presentation and viral persistence, while excessive TNF-α may paradoxically foster the pro-inflammatory milieu driving cervical carcinogenesis [206,208]. Because cervical carcinogenesis is overwhelmingly driven by persistent hrHPV infection, these cytokine variants are presumed to predominantly modulate immune responses against oncogenic HPV genotypes rather than lrHPV types.

IFN-γ is a type II interferon central to antiviral and antitumor immunity, macrophage activation, and CTL effector function. A functional +874T/A polymorphism (rs2430561) within the *IFN*-γ gene has been associated with altered cytokine production and cervical cancer susceptibility [209,210,211]. A meta-analysis involving approximately 2375 cervical cancer cases and 2106 controls demonstrated a significant association between the IFN-γ +874 T/A polymorphism and increased cervical cancer risk [209]. Although ethnic variation in IFN-γ allele distribution has been documented among South African women, direct associations with cervical cancer risk remain inconsistent across cohorts [207], underscoring the need for adequately powered, population-stratified immunogenetic studies throughout Africa.

An important consideration when interpreting cytokine polymorphism studies in HPV-associated disease is the marked heterogeneity among individual high-risk HPV (hrHPV) genotypes rather than simply the distinction between hrHPV and low-risk HPV. Most available immunogenetic studies have evaluated cervical cancer or persistent HPV infection as a composite hrHPV phenotype and therefore predominantly reflect the biology of the genotypes most represented in European and North American cohorts, principally HPV16 and HPV18, with comparatively fewer data for HPV31, HPV33, and HPV45 [200,201,202]. As discussed in Section 4.1, this genotype distribution differs substantially from that observed across sub-Saharan Africa, where HPV35, HPV52, and HPV58 contribute disproportionately to persistent infection and cervical cancer burden. Notably, HPV35 has been consistently detected across longitudinal African cohorts, including studies from Ethiopia and Rwanda, indicating that it represents a persistent component of the regional hrHPV landscape rather than a transient infection [212,213]. Despite its epidemiological importance, published evidence evaluating whether host cytokine polymorphisms including variants within *IL10*, *TNF-α*, and *IFNG* differentially influence persistence or clearance of HPV35, HPV52, or HPV58 remains extremely limited, and most studies continue to analyze these genotypes within pooled hrHPV categories. Consequently, whether high IL-10–producing or reduced IFN-γ–producing genotypes exert similar effects on HPV35 persistence as they do on HPV16 remains unknown. This represents an important knowledge gap for Africa, particularly because HPV35 is not directly targeted by current bivalent or quadrivalent vaccines and receives limited protection from existing vaccination strategies. Future immunogenetic studies should therefore incorporate genotype-stratified analyses, examining HPV35, HPV52, and HPV58 as distinct outcomes while accounting for important modifiers such as HIV coinfection, age, and other endemic co-infections. Such studies will be essential for determining whether host cytokine variation differentially influences viral persistence and cervical carcinogenesis across the HPV genotypes that predominate in African populations.

### 4.3. Clinical Implications of Immunogenetic Variation: Disease Progression, Therapeutic Response, and Modifying Factors

Section 4.1 and Section 4.2 demonstrate that host immunogenetic variation influences not only susceptibility to oncogenic viral infection, but also disease progression and clinical outcomes in African populations. Cytokine polymorphisms have been associated with hepatic fibrosis progression in HBV-infected Ghanaian patients [181], HLA-B*57 alleles distinguish elite HIV control from accelerated disease progression [174,175,176], IL-10 promoter variants influence cervical cancer risk beyond their effects on hrHPV persistence [201,202], and regulatory T-cell infiltration correlates with EBV DNA load and prognosis in nasopharyngeal carcinoma [214]. Building on these observations, this section examines two additional but less explored dimensions of immunogenetic diversity: its influence on therapeutic response and the population-level factors that modify or confound genotype–phenotype associations in African settings.

#### 4.3.1. Immunogenetic Determinants of Therapeutic Response

Host genetic variation can substantially influence responses to antiviral and antiretroviral therapies. The clearest example comes from HCV infection. In Egyptian cohorts, the IFNL3 (IL28B) rs12979860 CC genotype was associated with increased spontaneous viral clearance and improved response to pegylated interferon/ribavirin therapy [215,216,217]. Importantly, this association persists in the direct-acting antiviral era, with the CC genotype remaining associated with higher sustained virologic response rates following sofosbuvir/daclatasvir treatment [218]. Similarly, HLA-DRB1*13:02, previously linked to spontaneous HBV clearance in Gambian cohorts, has also been associated with favorable interferon treatment responses in chronic HBV infection [219], suggesting that common immunogenetic pathways may influence both natural and treatment-induced viral control.

By contrast, host genetic determinants of response to HBV nucleos(t)ide analog therapy remain poorly characterized in African populations. Although treatment failure is largely attributed to viral resistance mutations, including tenofovir-associated resistance mutations reported in South African HBV genotypes A and D [214,220,221], no African genome-wide or candidate-gene studies have specifically evaluated host predictors of treatment response. Given the major burden of HBV-related hepatocellular carcinoma across Africa, this represents an important knowledge gap.

Beyond nucleos(t)ide analog therapy, pegylated interferon-α (Peg-IFN-α) remains the only finite-duration therapy capable of achieving functional cure in chronic HBV infection, making host immune signaling pathways important determinants of therapeutic response. Peg-IFN-α exerts its antiviral activity through binding to the type I interferon receptor complex (IFNAR1/IFNAR2), activation of the JAK1–TYK2–STAT1/STAT2 signaling cascade, and induction of interferon-stimulated genes (ISGs) that establish an intracellular antiviral state [222]. Consequently, genetic variation affecting interferon receptor signaling, downstream JAK–STAT activation, or ISG induction may influence treatment responsiveness.

The IFNL3 (IL28B) locus provides one of the best-characterized examples of host pharmacogenetic regulation of interferon therapy; however, its relevance to Peg-IFN-α treatment in chronic HBV remains uncertain. An early study in HBeAg-positive patients reported a significant association between IFNL3 genotype and Peg-IFN-induced serological response [223], whereas a pooled analysis of 701 patients from three international clinical trials found no significant association in either HBeAg-positive or HBeAg-negative chronic HBV infection [224]. In addition, constitutively elevated baseline hepatic ISG expression has been associated with reduced interferon responsiveness, suggesting that host variation influencing ISG regulation may further contribute to treatment heterogeneity, although this mechanism has not been systematically investigated in African HBV cohorts [225]. This uncertainty is particularly relevant for Africa, where IFNL3 allele frequencies differ substantially from those reported in European and Asian populations [226,227], yet no study has examined whether these differences influence Peg-IFN responsiveness in African patients, who are predominantly infected with HBV genotype E. Prospective studies integrating IFNL3 genotype, IFNAR/JAK–STAT pathway variation, ISG expression profiles, viral genotype, and clinical treatment outcomes are therefore needed to determine whether host immunogenetic biomarkers can guide individualized Peg-IFN therapy in African populations.

Pharmacogenetic variation also influences antiretroviral therapy outcomes in HIV-positive individuals, indirectly affecting the risk of KSHV- and EBV-associated malignancies. Efavirenz and nevirapine metabolism is strongly influenced by CYP2B6 polymorphisms, variants of which occur at high frequency in many African populations [221,228]. Studies from Ethiopia, Tanzania, and Zimbabwe have demonstrated substantial population-level differences in efavirenz metabolism and drug clearance associated with CYP2B6 genotype [229,230]. Because effective HIV suppression and immune reconstitution reduce susceptibility to virus-associated cancers, these pharmacogenetic differences have important downstream implications for oncogenesis.

For HPV, EBV, KSHV, and HTLV-1-associated malignancies, African-specific pharmacogenetic data remain extremely limited. Host determinants of immunotherapy response in cervical cancer, rituximab response in EBV-associated lymphomas, and treatment outcomes in HTLV-1-associated malignancies have not been adequately studied in African cohorts [231]. Consequently, current pharmacogenetic evidence remains disproportionately concentrated on HCV and HIV treatment.

#### 4.3.2. Clinical and Population-Level Modifiers of Immunogenetic Associations

Interpretation of immunogenetic studies in Africa requires consideration of several important modifying factors, particularly HIV co-infection, age at infection, and population heterogeneity. HIV co-infection is a major modifier of both disease progression and therapeutic response. HIV substantially increases the risk of chronic HBV infection and invasive cervical cancer in HPV-infected women [232]. It may also alter vaccine effectiveness; for example, an HPV vaccine study in Rwanda reported lower effectiveness among HIV-positive compared with HIV-negative participants [233]. Failure to account for HIV status may therefore confound genotype–outcome associations.

For HBV infection, age at acquisition is an equally important determinant of outcome. Approximately 90% of perinatally infected individuals develop chronic infection, compared with fewer than 5% of adults infected later in life [234,235]. Because transmission patterns vary considerably across African regions, studies evaluating genetic determinants of viral persistence or clearance may be confounded if age at infection is not considered.

Finally, substantial genetic diversity exists across African populations. Allele frequencies for HLA loci, IFNL3/IFNL4, and CYP2B6 vary considerably between West, East, Central, and Southern African populations [221,228]. Consequently, findings derived from single-population studies should not be generalized across the continent without independent validation. Some inconsistencies observed across African immunogenetic studies may therefore reflect differences in HIV prevalence, transmission patterns, or population structure rather than true biological differences in genetic effects.

### 4.4. Integrative Perspectives: Immunogenetics in the African Cancer Landscape

Virus-associated cancers in Africa emerge within a uniquely complex immunological ecosystem shaped by the convergence of host genetic diversity, chronic infectious exposure, and persistent immune perturbation. The immunogenetic factors described above do not operate independently but rather interact dynamically with co-endemic infections, nutritional status, environmental exposures, and socioeconomic determinants to influence antiviral immunity and cancer susceptibility across African populations. Consequently, oncogenic viral persistence in Africa is best understood through a systems immunogenetics framework in which host genetics and environmental immune pressures collectively determine disease trajectory.

In many African settings, chronic exposure to malaria, helminths, HIV, and tuberculosis establishes a baseline immune environment characterized by persistent immune activation, regulatory polarization, and altered cytokine homeostasis. These conditions frequently promote IL-10-dominant immunoregulation, impaired IFN-γ-mediated antiviral responses, chronic antigenic stimulation, and progressive T cell dysfunction. Within this context, cytokine polymorphisms associated with elevated IL-10 production or dysregulated TNF-α and IFN-γ signaling may exert amplified biological effects, promoting viral persistence, immune evasion, and tumor-promoting inflammation. Likewise, chronic inflammatory signaling and repeated immune activation may accelerate immune exhaustion pathways that compromise long-term control of oncogenic viruses such as HPV, HBV, EBV, and KSHV.

Simultaneously, the extraordinary HLA diversity observed across African populations may confer both protective and susceptibility-associated effects on viral pathogenesis. Certain HLA alleles may enhance antigen presentation breadth and CTL responsiveness against oncogenic viral epitopes, whereas others may facilitate immune escape through suboptimal peptide presentation [236]. These effects are likely further modified by pathogen diversity, viral genotype distribution, and co-infection burden, creating substantial interpopulation heterogeneity in viral clearance, chronic infection, and cancer risk. Importantly, many immunogenetic associations identified in European and East Asian cohorts may not be directly transferable to African populations because of profound differences in allelic architecture and infectious exposure histories [236].

Collectively, these observations support the concept that virus-associated malignancies in Africa arise through multilayered interactions between host immunogenetics, chronic infectious exposures, and sustained immune dysregulation. Despite this complexity, African populations remain substantially underrepresented in large-scale immunogenomic studies of viral carcinogenesis. Future research integrating genome-wide association studies, high-resolution HLA sequencing, transcriptomics, cytokine profiling, and detailed co-infection phenotyping within prospective African cohorts will be essential for defining predictive biomarkers of cancer susceptibility and therapeutic responsiveness. Such efforts may ultimately facilitate the development of precision immunological strategies for vaccination, cancer prevention, and immunotherapy tailored to African epidemiological and genetic contexts.

## 5. Tumor Immune Microenvironment in Virus-Associated Cancers

**Figure 3:**
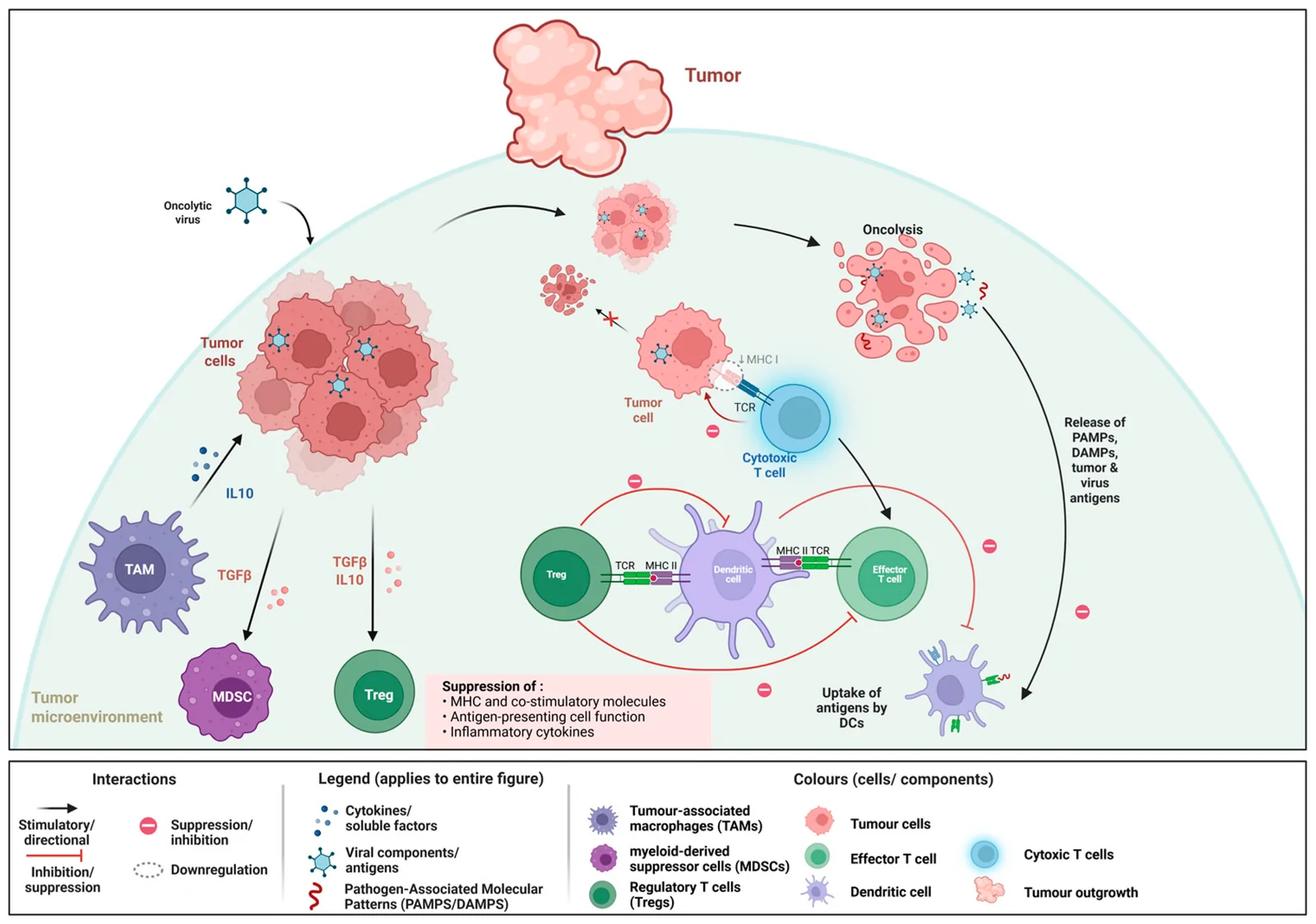
Immunosuppressive tumor microenvironment in virus-associated cancers. Schematic representation of the immunosuppressive tumor microenvironment (TME) in virus-associated malignancies. Tumor-associated macrophages (TAMs), myeloid-derived suppressor cells (MDSCs), and regulatory T cells (Tregs) establish a suppressive immune landscape through dominant IL-10 and TGF-β signaling, resulting in impaired dendritic cell (DC) activation, reduced antigen presentation, and diminished effector T-cell priming. Viral- and tumor-mediated downregulation of MHC class I and II molecules further compromise cytotoxic T-cell recognition and antiviral immune surveillance.

A defining hallmark of virus-associated malignancies is the progressive establishment of an immunosuppressive tumor microenvironment (TME) that promotes viral persistence, immune escape, and malignant progression (Figure 3). Although oncogenic viruses differ in cellular tropism and mechanisms of transformation, they frequently converge on common immunological pathways that suppress antiviral immunity while sustaining chronic inflammation and tumor survival. In African populations, where persistent exposure to infectious pathogens and chronic immune activation are highly prevalent, the tumor immune microenvironment is further shaped by repeated co-infections, inflammatory stress, and immune dysregulation. Consequently, the TME in virus-driven cancers represents not merely a passive consequence of transformation, but an active immunological niche that supports oncogenesis, viral latency, and therapeutic resistance.

### 5.1. The Immunosuppressive Tumor Milieu

One of the most consistent features of virus-associated tumors is the accumulation of suppressive immune cell populations [237,238], including regulatory T cells (Tregs), tumor-associated macrophages (TAMs), and myeloid-derived suppressor cells (MDSCs), which collectively inhibit effective antitumor immunity [239,240,241]. These cell populations are highly enriched in HPV-associated cervical cancer, EBV-associated nasopharyngeal carcinoma (NPC) and lymphomas, HBV/HCV-associated hepatocellular carcinoma (HCC), and Kaposi sarcoma, where they establish a profoundly tolerogenic microenvironment characterized by elevated IL-10 and transforming growth factor-β (TGF-β) signaling.

Rather than acting independently, these suppressive populations function as an integrated immunoregulatory network (Figure 3). Tregs are central mediators of immune suppression in virus-driven tumors. Through expression of FOXP3 and secretion of inhibitory cytokines including IL-10 and TGF-β, Tregs suppress the activation, proliferation, and cytotoxic activity of virus-specific CD8^+^ T cells and helper CD4^+^ T cells, thereby impairing immune-mediated clearance of infected or transformed cells [242,243]. Increased infiltration of CD4^+^CD25^+^FOXP3^+^ Tregs has been consistently associated with poor prognosis in EBV-associated NPC and HBV-related HCC [244,245,246,247,248]. In NPC, intratumoral Treg abundance correlates positively with plasma EBV DNA levels, suggesting that persistent viral antigen exposure actively reinforces local immune tolerance and facilitates immune escape [247].

TAMs constitute another dominant immunosuppressive population within virus-associated tumors. Although macrophages may adopt either classically activated M1 or alternatively activated M2 phenotypes, oncogenic viruses frequently skew macrophage polarization toward an M2-like, tumor-promoting state [249,250,251,252,253]. M2-polarized TAMs secrete IL-10, TGF-β, vascular endothelial growth factor (VEGF), arginase-1, and immune checkpoint ligands such as PD-L1, collectively suppressing cytotoxic T lymphocyte (CTL) and natural killer (NK) cell function while simultaneously promoting angiogenesis, fibrosis, invasion, and metastatic dissemination [254]. In HPV-driven cervical cancer, persistent viral infection promotes M2 polarization through chronic inflammatory signaling and suppression of innate sensing pathways, including cGAS–STING signaling, thereby facilitating immune evasion and therapeutic resistance [241,254].

Direct immunophenotypic characterization of TAM populations in African virus-associated cancers remains limited. Nevertheless, many patients with HBV-associated HCC and cervical cancer in Africa present with advanced-stage disease, suggesting prolonged periods of ineffective tumor immune surveillance and underscoring the need for studies directly profiling the tumor immune microenvironment in African populations [255].

MDSCs represent an additional layer of immune suppression in virally induced cancers. These immature myeloid populations, which include monocytic (M-MDSC) and polymorphonuclear (PMN-MDSC) subsets, expand in response to chronic inflammatory signaling and tumor-associated “emergency myelopoiesis” [239,256,257,258]. MDSCs suppress antitumor immunity through multiple mechanisms, including inhibition of T-cell proliferation, depletion of amino acids required for T-cell activation, reactive oxygen species generation, and suppression of NK-cell cytotoxicity.

Evidence from African populations demonstrates that suppressive myeloid responses can be established early in life in settings characterized by chronic infectious exposures. In a South African pediatric cohort, monocytic MDSC-like populations were significantly expanded in HIV-exposed and HIV-infected children with tuberculosis compared with unexposed controls, and these cells suppressed IL-12 and TNF-α production in response to mycobacterial antigens [259]. Although these observations were not made in cancer patients, they suggest that chronic infectious exposures common in many African settings may promote systemic immunoregulatory states that could influence antitumor immunity.

In virus-associated cancers such as cervical cancer, HCC, Kaposi sarcoma, and EBV-associated lymphomas, MDSCs reinforce local immune paralysis through production of IL-10, TGF-β, inducible nitric oxide synthase (iNOS), and arginase-1 [256,260,261]. Persistent endoplasmic reticulum (ER) stress signaling, particularly through the IRE1α and ATF6 branches of the unfolded protein response (UPR), further enhances the suppressive activity of PMN-MDSCs within tumors [257]. Importantly, this molecular pathway may also provide a mechanistic link between the regional co-infection syndemics discussed in Section 3 and the establishment of an immunosuppressive tumor microenvironment. Chronic infections that are highly prevalent across Africa, including HIV and *Plasmodium falciparum* malaria, have each been shown to activate UPR signaling through ER stress induced by sustained inflammatory stimulation, oxidative stress, hypoxia, and increased protein-folding demand [262,263]. Activation of the IRE1α pathway promotes NF-κB signaling and inflammatory transcriptional programs that influence myeloid-cell differentiation and function, whereas persistent UPR activation has been implicated in enhancing the suppressive phenotype of tumor-associated PMN-MDSCs [257]. Although direct evidence from African virus-associated cancers is currently lacking, these observations suggest that chronic systemic inflammation associated with HIV and malaria may lower the threshold for ER stress-mediated MDSC activation within developing tumors, thereby linking endemic infectious exposures with local immune suppression. This hypothesis warrants investigation in prospective African cohorts integrating co-infection status, ER stress biomarkers, and tumor immune profiling. Therapeutically, strategies aimed at depleting MDSCs, blocking their recruitment, or reprogramming them toward mature myeloid phenotypes are increasingly being explored in combination with immune checkpoint blockade in virally driven cancers [256,260,261].

These suppressive cellular networks establish a cytokine milieu dominated by IL-10 and TGF-β, which profoundly dampens antiviral and antitumor immunity (Figure 3). IL-10 suppresses dendritic cell maturation, inhibits antigen presentation, and reduces production of pro-inflammatory cytokines necessary for T-cell priming. TGF-β exerts broad inhibitory effects on CTLs and NK cells while simultaneously promoting epithelial–mesenchymal transition, fibrosis, extracellular matrix remodeling, and metastatic progression. Together, these pathways shift the balance away from immune surveillance and toward immune tolerance, allowing persistent viral infection and malignant evolution to proceed simultaneously.

### 5.2. Viral and Tumor-Mediated Disruption of Antigen Presentation

Effective antiviral and antitumor immunity depends on the presentation of viral and tumor-derived peptides by major histocompatibility complex (MHC) molecules to CD8^+^ and CD4^+^ T lymphocytes (Figure 3). Consequently, disruption of antigen presentation represents one of the most important mechanisms by which oncogenic viruses evade immune surveillance and establish persistent infection. Although the molecular mechanisms underlying antigen-processing defects have been largely characterized in non-African populations and experimental systems, emerging evidence suggests that these pathways may have particular relevance in Africa, where extensive HLA diversity, high pathogen exposure, and chronic immune activation influence host–pathogen interactions.

Several oncogenic viruses directly interfere with antigen presentation pathways. HPV E5 reduces surface expression of MHC class I molecules by retaining HLA complexes within the Golgi apparatus, thereby limiting recognition of infected epithelial cells by cytotoxic T lymphocytes (CTLs) [264]. Similarly, EBV latent proteins modulate antigen-processing pathways and restrict the presentation of viral epitopes during latency, enabling infected B cells to evade immune elimination [265]. KSHV further suppresses antigen presentation through viral proteins that promote degradation or intracellular sequestration of MHC class I molecules, while chronic HBV infection is associated with impaired hepatocyte antigen presentation and progressive dysfunction of antiviral T-cell responses [266,267].

The importance of intact antigen presentation in African populations is supported by genetic studies of endemic Burkitt lymphoma (eBL). As discussed in Section 4.1.1, a high-resolution HLA study involving children from northern Uganda demonstrated that HLA-A02 and HLA-B58, alleles associated with efficient presentation of EBV-derived epitopes, were significantly less frequent among eBL cases than controls, whereas HLA-DQA1 homozygosity independently increased disease risk [177]. These findings provide direct population-level evidence that host genetic variation affecting antigen presentation influences susceptibility to EBV-driven malignancy in African populations.

Beyond direct viral effects, tumor evolution imposes additional selective pressures favoring immune escape. Virus-associated cancers frequently acquire genetic and epigenetic alterations affecting key components of the antigen-processing machinery (APM), including HLA heavy chains, β2-microglobulin, TAP1/2, tapasin, ER aminopeptidases (ERAP1/2), and immunoproteasome subunits [264,265,266,267]. Loss or dysfunction of these molecules limits the presentation of both viral antigens and tumor neoantigens, thereby reducing susceptibility to CTL-mediated killing. Altered interferon signaling may further reinforce this immune invisibility. Suppression of interferon regulatory factors, particularly IRF2, reduces APM expression while simultaneously promoting PD-L1 upregulation, effectively coupling defective antigen presentation with active immune suppression.

Despite the central role of antigen presentation in antiviral immunity, direct characterization of antigen-processing defects in African virus-associated cancers remains limited. Most mechanistic studies have been performed in cell lines or cohorts from outside Africa, and it remains unclear whether the extensive HLA diversity observed across African populations modifies patterns of immune escape, tumor evolution, or responses to immunotherapy. Addressing these knowledge gaps will be essential for understanding regional differences in virus-associated cancer susceptibility and for optimizing immune-based therapeutic strategies on the continent.

### 5.3. Dendritic Cell Dysfunction and Failure of T-Cell Priming

Although antigen presentation by infected cells is critical for immune recognition, effective antitumor immunity ultimately depends upon dendritic cells (DCs), which serve as the principal initiators of adaptive immune responses. Through antigen uptake, processing, and cross-presentation, DCs activate naïve CD4^+^ and CD8^+^ T lymphocytes and orchestrate the generation of durable antiviral immunity. In virus-associated cancers, however, DC function is profoundly impaired, resulting in defective T-cell priming and inadequate immune surveillance [268,269]. Multiple factors within the tumor microenvironment contribute to DC dysfunction. Elevated concentrations of IL-10, TGF-β, vascular endothelial growth factor (VEGF), prostaglandin E2, and other suppressive mediators inhibit dendritic cell maturation and prevent acquisition of an immunostimulatory phenotype [268,269]. Consequently, DCs exhibit reduced expression of MHC-II molecules, co-stimulatory molecules such as CD80, CD86, and CD83, and cytokines required for T-cell activation, including IL-12 [270].

Virus-associated tumors further promote the accumulation of immature or tolerogenic DC populations that preferentially induce T-cell anergy and regulatory T-cell differentiation rather than effective antiviral responses [271]. In HPV-associated cervical cancer and HBV-related HCC, reduced dendritic cell density within tumors has been correlated with impaired CTL infiltration and poor clinical outcome [272]. Similar observations have been reported in EBV-associated malignancies, where persistent viral antigen exposure contributes to functional exhaustion of antigen-presenting cells [154]. Hypoxia, metabolic stress, and nutrient competition within the tumor microenvironment further compromise DC function [273]. Tumor cells and suppressive myeloid populations consume essential metabolites required for immune activation, creating a metabolically hostile environment that restricts dendritic cell survival and antigen-presenting capacity [274]. Consequently, even when substantial quantities of tumor-derived or viral antigens are released during cell death, these antigens frequently fail to generate productive adaptive immune responses. Failure of dendritic cell priming therefore represents a critical link between local immune suppression and systemic immune dysfunction. Without effective DC activation, the generation of robust antiviral and antitumor T-cell responses becomes severely compromised, allowing persistent viral infection and malignant progression to proceed unchecked.

### 5.4. Immune Checkpoint Signaling and T-Cell Exhaustion

**Figure 4:**
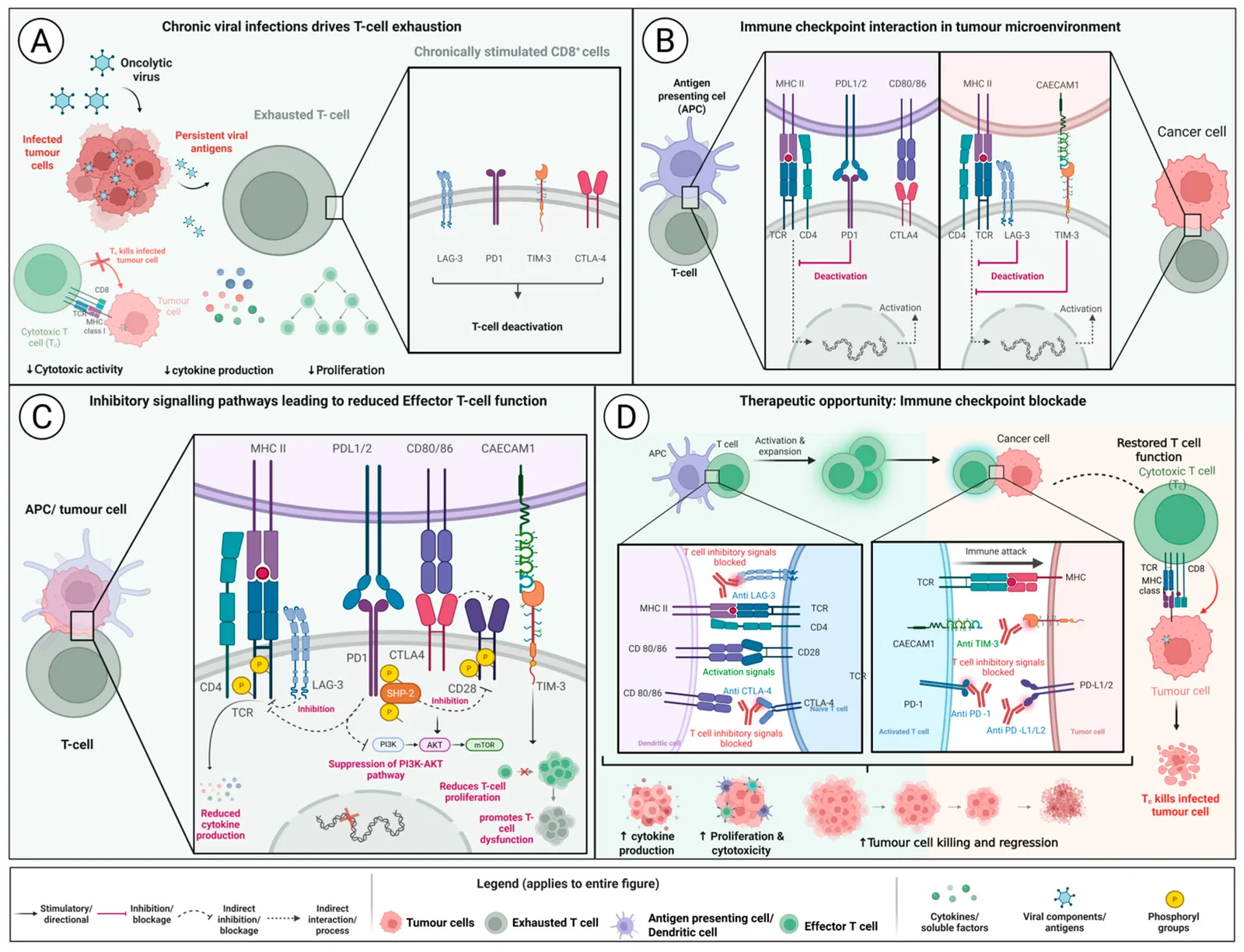
Immune checkpoint signaling and therapeutic restoration of antiviral T-cell function in virus-associated cancers. Panel (**A**). Chronic viral antigen exposure within virus-associated tumors promotes progressive CD8^+^ T-cell exhaustion, characterized by reduced cytotoxic activity, diminished cytokine production, impaired proliferation, and increased expression of inhibitory receptors, including PD-1, CTLA-4, TIM-3, and LAG-3. Panel (**B**). Immune checkpoint interactions between exhausted T cells, antigen-presenting cells (APCs), and tumor cells suppress T-cell activation through inhibitory receptor–ligand signaling within the tumor microenvironment. Panel (**C**). Downstream inhibitory signaling mediated by PD-1, CTLA-4, TIM-3, and LAG-3 suppresses PI3K–AKT–mTOR signaling, reduces cytokine production and proliferation, and promotes T-cell dysfunction and effector exhaustion. Panel (**D**). Immune checkpoint blockade restores antitumor immunity by disrupting inhibitory signaling pathways, thereby enhancing T-cell activation, proliferation, cytokine production, cytotoxic function, and tumor cell killing, ultimately promoting tumor regression.

Persistent exposure to viral antigens is a defining characteristic of chronic oncogenic viral infections and represents a major driver of T-cell exhaustion (Figure 4A) [275,276]. Unlike acute infections, where antigen clearance results in the formation of functional memory responses, chronic antigenic stimulation induces a progressive state of T-cell dysfunction characterized by impaired proliferation, reduced cytokine production, diminished cytotoxicity, and sustained expression of inhibitory immune checkpoint receptors [277,278].

Among the most extensively studied checkpoint pathways are programmed cell death protein-1 (PD-1) and cytotoxic T lymphocyte-associated antigen-4 (CTLA-4) (Figure 4B). Engagement of PD-1 by its ligand PD-L1 suppresses T-cell receptor signaling, inhibits cytokine production, and reduces metabolic fitness [279,280], whereas CTLA-4 competes with CD28 for co-stimulatory ligands and attenuates early T-cell activation [281]. Additional inhibitory receptors, including TIM-3 and LAG-3, are frequently co-expressed on exhausted T cells and contribute to profound immune dysfunction during chronic viral infection [282].

Checkpoint dysregulation has now been documented in several African virus-associated malignancies. In a large cohort of cervical squamous cell carcinoma from Mozambique, PD-L1 expression was detected in approximately one-quarter of tumors, providing direct evidence that checkpoint-mediated immune suppression is common in HPV-associated cervical cancer in HIV-endemic African settings [283]. Interestingly, PD-L1 expression did not differ significantly according to HIV status, suggesting that the relationship between HIV-associated immune dysregulation and checkpoint activation may be more complex than previously assumed [283]. Similarly, a Nigerian cervical cancer cohort demonstrated substantial heterogeneity in PD-L1 expression despite a high prevalence of oncogenic HPV genotypes, indicating that checkpoint activation may vary considerably across African populations and tumor contexts [284]. In Kaposi sarcoma, strong expression of PD-1, PD-L1, and PD-L2 has been demonstrated in tumor tissues from HIV-positive patients, supporting a biological rationale for immune checkpoint blockade in KSHV-associated malignancies, which remain highly prevalent across sub-Saharan Africa [276,285].

At the molecular level, checkpoint receptor engagement activates multiple inhibitory signaling pathways that suppress T-cell receptor signaling, impair cellular metabolism, and reduce effector gene transcription [286] (Figure 4C). These alterations collectively reinforce the exhausted phenotype and limit effective antiviral immunity. Virus-associated tumors actively exploit checkpoint pathways to evade immune elimination [287]. EBV-driven malignancies frequently exhibit high levels of PD-L1 expression [288], while KSHV-associated tumors establish chronically suppressive environments that sustain checkpoint signaling and prevent effective immune clearance [289]. Consequently, exhausted T cells remain present within tumors but are functionally incapable of controlling either viral replication or malignant growth [290]. Importantly, the exhausted state is not entirely irreversible, and therapeutic interruption of checkpoint signaling can partially restore antiviral and antitumor immune function [291,292] (Figure 4D). The emergence of immune checkpoint blockade has transformed cancer immunotherapy by demonstrating that exhausted T cells can be partially reinvigorated through interruption of inhibitory signaling pathways [292,293]. Therapeutic antibodies targeting PD-1, PD-L1, and CTLA-4 have shown efficacy across multiple virus-associated malignancies and represent a promising strategy for restoring antiviral immunity [294,295]. However, the impact of extensive HLA diversity, chronic co-infections, and unique immunological exposures characteristic of African populations remains poorly understood and warrants further investigation.

### 5.5. Immunotherapeutic Opportunities and Challenges in African Populations

The growing understanding of immune evasion mechanisms in virus-associated cancers has created new opportunities for immunotherapeutic intervention. As illustrated in Figure 4D, immune checkpoint blockade can partially reverse T-cell exhaustion by restoring effector function, cytokine production, and antitumor activity [296,297]. Clinical studies have demonstrated the efficacy of inhibitors targeting PD-1, PD-L1, and CTLA-4 in several virus-associated malignancies, including HPV-associated cervical cancer, HBV-related hepatocellular carcinoma, EBV-associated nasopharyngeal carcinoma, and Kaposi sarcoma [298]. The continued expression of viral antigens within many of these tumors provides attractive immunological targets and may enhance responsiveness to immune-based therapies.

Beyond checkpoint inhibition, therapeutic cancer vaccines and adoptive cellular therapies offer additional strategies for harnessing antiviral immunity [299]. Vaccines targeting HPV E6/E7 oncoproteins, EBV latent antigens, and HBV-associated tumor antigens aim to stimulate antigen-specific T-cell responses, while virus-specific T-cell therapies have demonstrated encouraging results in selected EBV-associated malignancies [300]. Collectively, these approaches highlight the therapeutic potential of exploiting viral antigens that persist throughout tumor development.

Despite this strong biological rationale, major challenges limit the implementation of immunotherapy across Africa. African populations remain markedly underrepresented in immunotherapy clinical trials; less than 1% of international cancer drug registration trials submitted to the US FDA include participants from African clinical sites, despite the continent comprising more than 18% of the global population [301,302]. This underrepresentation has delayed the generation of context-specific evidence. For example, rituximab, approved in the United States in 1997 for diffuse large B-cell lymphoma, was not prospectively evaluated in a dedicated African clinical trial until 24 years later, when a rituximab biosimilar study was conducted in Malawi [303].

In addition, extensive HLA diversity, high burdens of HIV, malaria, tuberculosis, and helminth infections, and substantial variation in immune activation states may influence treatment responsiveness and toxicity. Furthermore, African populations remain markedly underrepresented in immunotherapy clinical trials, limiting understanding of how regional immunological and genetic factors affect therapeutic outcomes. Limited access to molecular diagnostics, biomarker testing, and high-cost biologic therapies further constrains the widespread adoption of precision immuno-oncology across many settings. Consequently, the applicability of immunotherapeutic strategies developed largely in European, North American, and Asian populations cannot be assumed for African populations without direct evaluation.

Addressing these challenges will require greater inclusion of African populations in translational research and clinical trials, together with expanded investment in genomic surveillance, molecular diagnostics, and cancer immunology research. Such efforts will be essential for developing context-specific immunotherapeutic strategies and ensuring equitable access to emerging treatments for virus-associated cancers across Africa.

## 6. Public Health Gaps and Policy Implications

### 6.1. Limitations in Screening, Early Detection, and Diagnostic Infrastructure

Early detection remains one of the most effective interventions for reducing mortality associated with virus-driven malignancies; however, diagnostic and screening infrastructures across much of Africa remain insufficient to support timely identification of precancerous or early-stage disease. Persistent infection with high-risk Human papillomavirus (hrHPV) genotypes underlies the majority of cervical cancer cases globally, yet population-level screening coverage across many African countries remains critically low [2,304]. Organized national screening programs are often absent, and opportunistic screening strategies dominate, leading to delayed identification of precancerous lesions and advanced-stage diagnoses. These limitations contribute substantially to the high cervical cancer mortality rates observed across the region. Diagnostic gaps also affect HCC, which is strongly associated with chronic infection by HBV and HCV. Although surveillance strategies such as ultrasound imaging and alpha-fetoprotein testing are recommended for high-risk individuals, these tools are not consistently available across many healthcare systems in Africa [296,305]. Limited access to diagnostic imaging technologies, shortages of trained specialists, and constrained healthcare infrastructure frequently result in late-stage diagnosis when curative treatment options are no longer feasible. In addition to cervical and liver cancers, malignancies associated with viruses such as EBV and KSHV remain challenging to diagnose in resource-limited settings. Detection of these viruses often requires molecular assays or specialized histopathological techniques that are not widely available [1]. Strengthening laboratory capacity and expanding molecular diagnostic infrastructure will therefore be essential for improving early detection and clinical management of virus-associated cancers across the continent.

Addressing these deficiencies will require coordinated efforts from multiple stakeholders. National governments and ministries of health must prioritize investment in cancer screening programs, diagnostic infrastructure, and workforce development while integrating virus-associated cancer surveillance into broader public health strategies. Regional and international public health agencies can support the establishment of standardized screening guidelines, cancer registries, and surveillance networks to improve disease monitoring and resource allocation. Academic and research institutions play a critical role in strengthening local diagnostic capacity, training healthcare professionals, and generating context-specific evidence to inform policy and clinical practice. In parallel, international funding organizations, development partners, and philanthropic agencies must provide sustained financial support for laboratory infrastructure, molecular diagnostics, and implementation research aimed at expanding access to early detection services. Put together, these efforts will be essential for building resilient diagnostic systems capable of reducing delays in diagnosis and improving outcomes for patients with virus-associated cancers across Africa.

### 6.2. Gaps in Preventive Vaccination and Population-Level Protection

Vaccination remains one of the most effective strategies for preventing virus-associated cancers [296,305]. Prophylactic HPV vaccination has demonstrated remarkable success in reducing infections with oncogenic HPV genotypes driving cervical carcinogenesis and is expected to further reduce future cervical cancer incidence [297,306]. Despite this promise, HPV vaccine coverage across Africa remains uneven, because of persistent challenges related to vaccine availability, healthcare infrastructure, funding constraints, and access to adolescent populations.

Preventive efforts targeting Hepatitis B virus have also achieved important progress, yet important gaps remain. While the HBV vaccine has been incorporated into routine childhood immunization schedules in many African countries, timely administration of the birth dose vaccine remains inconsistent [307]. This dose is essential for preventing vertical transmission, which represents a major route of chronic HBV infection in highly endemic regions. Structural and socioeconomic barriers further limit vaccination coverage. In addition to these barriers, vaccine uptake may be hindered by poor public awareness, limited access to reliable health information, and the growing influence of misinformation and disinformation [308]. Across several African countries, misconceptions regarding the HPV vaccine have contributed to reduced vaccine acceptance, including claims that vaccination causes infertility, promotes sexual promiscuity, serves as a population-control strategy, or poses significant safety risks despite extensive evidence demonstrating its safety and effectiveness [308]. Similar concerns have affected acceptance of other vaccination programs and may be amplified by social media platforms, inadequate risk communication, and mistrust of governmental or healthcare institutions [308,309]. Furthermore, reliance on traditional medicine and cultural beliefs regarding disease causation may influence perceptions of vaccine necessity and efficacy in some communities. Geographic inequalities in healthcare access, inadequate cold-chain infrastructure, and limited healthcare workforce capacity further undermine immunization efforts. Expanding immunization programs, strengthening vaccine delivery systems, improving public health communication, and fostering community engagement through culturally appropriate education campaigns will be central to increasing vaccine confidence and achieving sustainable reductions in the burden of virus-associated cancers across Africa.

### 6.3. Therapeutic Access and Translational Challenges

Despite major global advances in cancer immunotherapy and precision, access to these innovations remains extremely limited across many African healthcare systems. Immune checkpoint inhibitors targeting PD-1/PD-L1 and CTLA-4 pathways have demonstrated promising results in several virus-associated cancers, including cervical cancer linked to HPV and lymphomas associated with EBV [310]. However, the high cost of these therapies, limited oncology infrastructure, and lack of specialized treatment centers significantly restrict their availability in many African countries.

A further challenge is the underrepresentation of African populations in global cancer genomics and immunotherapy research. Many clinical trials evaluating novel cancer therapies are conducted primarily in North America, Europe, or Asia, leaving substantial gaps in knowledge regarding treatment responses among African populations [2]. Given the extraordinary genetic diversity of African populations and the high prevalence of chronic co-infections that shape immune function, findings derived from non-African populations may not fully capture determinants of disease susceptibility or therapeutic responsiveness across the continent.

Progress toward precision oncology is also constrained by limited genomic surveillance of oncogenic viruses and inadequate sequencing and bioinformatics capacity across many African countries [311]. Strengthening genomic surveillance, expanding molecular pathology services, increasing access to next-generation sequencing technologies, and improving regional bioinformatics expertise will be essential for identifying circulating viral variants, monitoring emerging resistance mechanisms, and supporting biomarker-driven therapeutic strategies. Expanding African participation in translational research and multicenter clinical trials will likewise be critical to ensuring that future immunotherapies and precision medicine approaches are applicable to the populations that bear the greatest burden of virus-associated cancers.

### 6.4. Future Research Priorities in Immunogenetics and Precision Oncology

Beyond the public health and policy priorities discussed above, several scientific and translational research gaps must be addressed to advance precision prevention and treatment of virus-associated cancers in Africa. Although accumulating evidence indicates that host immunogenetic variation influences viral persistence, disease progression, and therapeutic response, most available data originate from Asian, European, or relatively small African cohorts. Given the exceptional genetic diversity and distinct linkage disequilibrium structure of African populations, adequately powered multicountry studies are needed to identify population-specific determinants of susceptibility, immune regulation, and clinical outcomes [164,242]. Building on initiatives such as the Human Heredity and Health in Africa (H3Africa) consortium, future genome-wide association studies integrating HLA variation, cytokine polymorphisms, innate immune genes, viral genomics, and longitudinal clinical outcomes will provide a stronger foundation for precision oncology across the continent [312,313].

Future immunogenetic studies should also incorporate greater biological resolution by accounting for viral genotype diversity and key epidemiological modifiers. Many existing studies evaluate host polymorphisms using broad outcomes such as “high-risk HPV infection” or chronic viral hepatitis, despite substantial differences in oncogenic potential among viral genotypes. For example, no published African study has determined whether IL-10 or TNF-α promoter polymorphisms differentially influence persistence or clearance of HPV35, HPV52, or HPV58 relative to HPV16 or HPV18, despite the disproportionate contribution of these genotypes to cervical cancer across sub-Saharan Africa [14,18]. Comparable genotype-stratified investigations are needed for HBV, HCV, EBV, KSHV, and HTLV-1. Future studies should likewise account for HIV co-infection, malaria, helminth infections, age at infection, and regional population structure, all of which may substantially modify immunogenetic associations and contribute to inconsistent findings between African cohorts.

An equally important priority is expanding research into the pharmacogenomics of antiviral and immunomodulatory therapies. Although IFNL3 (IL28B) polymorphisms have been extensively investigated in relation to HCV treatment response, evidence regarding host genetic determinants of therapeutic response for chronic HBV remains limited and inconsistent. Early studies suggested an association between IFNL3 variants and pegylated interferon-induced HBeAg seroconversion, whereas subsequent pooled analyses failed to demonstrate a significant effect [223,224]. Moreover, no study has systematically evaluated IFNL3, JAK-STAT pathway components, interferon-stimulated gene (ISG) polymorphisms, or other immune regulatory loci as predictors of antiviral response specifically in African patients, despite marked differences in allele frequencies and the predominance of HBV genotype E across West Africa [314]. Similar evidence gaps exist regarding genetic predictors of response to immune checkpoint inhibitors, therapeutic cancer vaccines, adoptive cellular therapies, and other emerging immunotherapies for HPV-, EBV-, and KSHV-associated malignancies.

Addressing these priorities will require prospective, longitudinal cohort studies that integrate host immunogenetics, viral genomics, immune profiling, co-infection status, environmental exposures, and long-term clinical outcomes. The predominantly cross-sectional designs that characterize much of the current literature cannot adequately distinguish causal genetic effects from the influence of endemic co-infections and environmental factors that shape immune function across Africa. Integrating immunogenetic research into expanding vaccination programs, screening initiatives, genomic surveillance, and clinical trial networks represents the most effective strategy for generating evidence that can guide precision prevention, biomarker discovery, and individualized therapy for virus-associated cancers.

Ultimately, reducing the disproportionate burden of virus-associated cancers across Africa will require coordinated investment in prevention, early diagnosis, translational research, precision medicine, and equitable access to emerging therapies. Aligning advances in immunology, virology, genomics, and oncology with Africa-specific epidemiological and healthcare priorities will not only improve outcomes across the continent but will also provide globally relevant insights into the mechanisms of viral carcinogenesis and cancer immunology.

## 7. Conclusions

Virus-associated cancers represent one of the largest preventable contributors to the cancer burden in Africa. Although human papillomavirus (HPV), hepatitis B virus (HBV), hepatitis C virus (HCV), Epstein–Barr virus (EBV), Kaposi sarcoma-associated herpesvirus (KSHV), and human T-lymphotropic virus type 1 (HTLV-1) employ distinct strategies of infection and persistence, they converge on common immunological pathways that promote chronic inflammation, immune evasion, impaired antiviral surveillance, and malignant transformation. As summarized in Table 2, the progression from persistent viral infection to cancer is driven by a complex interplay among viral immune-evasion mechanisms, host immunogenetic diversity, endemic co-infections, chronic immune dysregulation, and evolution of an immunosuppressive tumor microenvironment.

This review highlights that Africa represents not only the region with the greatest burden of virus-associated malignancies but also a uniquely informative setting for understanding the immunology of viral carcinogenesis. The convergence of HIV, malaria, tuberculosis, and helminth infections with exceptional HLA and cytokine genetic diversity creates immunological environments that profoundly influence viral persistence, disease progression, and therapeutic responsiveness. These interactions underscore the importance of interpreting host genetic associations and immune mechanisms within their specific epidemiological and population contexts rather than extrapolating findings from non-African populations.

Major advances in prophylactic vaccination, antiviral therapy, cancer immunotherapy, and precision oncology have created unprecedented opportunities to reduce the burden of virus-associated cancers. However, these advances have not been equitably translated across much of Africa because of persistent limitations in vaccination coverage, screening programs, diagnostic infrastructure, genomic surveillance, access to emerging therapies, and participation in clinical and translational research. Addressing these disparities will require coordinated investments in public health systems alongside expansion of African-led immunology, genomics, and implementation research.

Ultimately, understanding how viral factors, host immunity, co-infections, and environmental exposures interact to shape carcinogenesis will be essential for developing effective prevention, diagnostic, and therapeutic strategies. Continued investment in multidisciplinary and multicountry research integrating virology, immunology, genomics, oncology, and public health will not only improve outcomes for African populations but will also generate fundamental insights into viral carcinogenesis that are broadly applicable to global cancer prevention and precision medicine.

## Figures and Tables

**Table 1:** Major oncogenic and oncogenesis-associated viral pathogens in Africa: cellular tropism, immune evasion mechanisms, and cancer associations.

Virus	Classification/Genome Type	Primary Cellular Targets	Persistence Mechanisms	Key Viral Oncogenes or Effector Proteins	Immune Evasion Strategies and Affected Host Pathways	Associated Malignancies	Oncogenic Co-Factors	References
High-risk human papillomavirus (hrHPV)	*Papillomavirus*; non-enveloped circular dsDNA virus	Basal epithelial cells of the cervical transformation zone, anogenital mucosa and oropharyngeal epithelium	Episomal persistence in basal keratinocytes; viral genome integration during progression; sustained E6/E7 expression	E6, E7, E5	E6-mediated p53 degradation; E7-mediated RB inactivation; impaired antigen presentation, interferon signaling, mucosal innate defense and tumor immune recognition	Cervical cancer; anal, vulvar, vaginal, penile and oropharyngeal cancers	HIV-associated immunodeficiency, persistent hrHPV infection, high-risk HPV genotype diversity (HPV16/18/35/45), limited screening, altered vaginal microbiome, smoking, reproductive and socioeconomic factors	[8,13,17,35]
Hepatitis B virus (HBV)	*Hepadnavirus*; enveloped partially double-stranded DNA virus with reverse transcription	Hepatocytes	Stable nuclear cccDNA reservoir; HBV DNA integration; chronic immune-tolerant or immune-exhausted infection	HBx, HBs/preS/S variants; insertional mutagenesis from HBV integration	Suppression of innate antiviral signaling; impaired interferon responses; T-cell exhaustion; chronic hepatic inflammation; altered antigen presentation and DNA-damage control	Hepatocellular carcinoma, frequently occurring at younger ages in high-endemicity African settings	Perinatal and early-childhood infection, HBsAg positivity, aflatoxin B1 exposure, cirrhosis, alcohol use, HCV/HIV coinfection	[10,56,109,110]
Hepatitis C virus (HCV)	*Flavivirus*; enveloped positive sense ssRNA virus	Hepatocytes	Chronic infection through quasispecies diversity, immune escape and persistent hepatic inflammation	No classical integrating oncogene; tumor-promoting proteins include core, NS3 and NS5A	Inhibition of RIG-I/MAVS and TLR3-linked interferon signaling; oxidative stress; chronic inflammation; fibrosis; T-cell dysfunction and exhaustion	Hepatocellular carcinoma, particularly in Egypt and parts of North Africa with historically high HCV prevalence	Cirrhosis, alcohol use, metabolic dysfunction, HBV/HIV coinfection, aflatoxin exposure	[39,60,111]
Epstein–Barr virus (EBV/HHV-4)	*Gammaherpesvirus*; enveloped linear dsDNA virus	B cells via CD21/CR2; epithelial cells in selected tissues	Lifelong latency in memory B cells; episomal genome maintenance; latency programs with restricted viral antigen expression	EBNA1, EBNA2, LMP1, LMP2A/B, EBERs, BART miRNAs	Reduced antigen processing and presentation; EBNA1 immune shielding; viral miRNA-mediated immune modulation; LMP1/LMP2 mimic B-cell survival signaling; dysregulation of NF-κB, JAK/STAT, apoptosis and B-cell activation	Endemic Burkitt lymphoma, Hodgkin lymphoma, diffuse large B-cell lymphoma, NK/T-cell lymphoma and nasopharyngeal carcinoma	*Plasmodium falciparum* malaria, HIV-associated immunosuppression, early-life EBV infection, MYC translocation, AID-driven genomic instability, HLA variation	[12,62,64,66]
Kaposi sarcoma-associated herpesvirus (KSHV/HHV-8)	*Gammaherpesvirus*; enveloped linear dsDNA virus	Endothelial and spindle cells; B cells; monocytes/macrophages	Latency with episomal maintenance by LANA; intermittent lytic reactivation; paracrine inflammatory and angiogenic signaling	LANA, vFLIP, vCyclin, vIL-6, vGPCR, K1, K15, viral miRNAs	Inhibition of apoptosis; NF-κB activation; downregulation of antigen presentation; interference with type I interferon pathways; cytokine mimicry; pro-angiogenic and immunosuppressive signaling	Endemic and HIV-associated Kaposi sarcoma; primary effusion lymphoma; multicentric Castleman disease	HIV infection, immunosuppression, chronic inflammation, high regional KSHV seroprevalence, coinfections	[75,76,77]
Human T-cell leukemia virus type 1 (HTLV-1)	*Deltaretrovirus*; enveloped positive sense ssRNA retrovirus with proviral DNA intermediate	Primarily CD4+ T cells, including regulatory T-cell-like populations	Lifelong proviral integration; persistence mainly through clonal expansion of infected T cells; low-level viral expression limiting immune recognition	Tax, HBZ, p12, p30	Intermittent Tax expression; HBZ-driven proliferation; dysregulation of NF-κB, cell-cycle control, DNA repair, CTLA-4/PD-1-associated immune regulation and T-cell survival	Adult T-cell leukemia/lymphoma in HTLV-1 endemic African regions	Breastfeeding transmission, sexual transmission, blood exposure, host genetics, long latency and immune dysregulation	[88,89,106]
Human immunodeficiency virus type 1 (HIV-1)	*Lentivirus*; enveloped positive sense ssRNA retrovirus with proviral DNA intermediate	CD4+ T cells, macrophages and dendritic cells	Latent proviral reservoirs; chronic immune activation; incomplete immune recovery despite viral suppression in some individuals	No dominant direct transforming oncogene; indirect oncogenesis through immunodeficiency, chronic inflammation and impaired control of oncogenic coinfections	CD4+ T-cell depletion; impaired CD8+ T-cell and NK-cell surveillance; chronic B-cell activation; reactivation or persistence of EBV, KSHV, HPV, HBV and HCV; altered HLA-restricted antiviral immunity	Kaposi sarcoma, cervical cancer, non-Hodgkin lymphoma, Hodgkin lymphoma, anal cancer and liver cancer in coinfected populations	KSHV, EBV, HPV, HBV/HCV coinfection, delayed ART initiation, low CD4 count and incomplete immune reconstitution	[112,113]

**Table 2:** Integrated immunological determinants of virus-associated cancers in Africa.

Determinant Category	Specific Factor	Immunological Effect	Virus(es) Affected	Cancer Outcome
Co-infection	HIV	CD4 depletion and immune exhaustion	HPV, EBV, KSHV, HBV	Cervical cancer, Kaposi sarcoma, lymphoma
Co-infection	Malaria	Reduced EBV-specific CTL responses	EBV	Burkitt lymphoma
Co-infection	Tuberculosis	Chronic inflammatory signaling	HBV, HCV	HCC
Host genetics	HLA polymorphisms	Altered antigen presentation	Multiple	Differential susceptibility
Host genetics	Cytokine polymorphisms	Altered immune regulation	Multiple	Variable cancer risk
Tumor microenvironment	Tregs	CTL suppression	Multiple	Tumor progression
Tumor microenvironment	TAMs/MDSCs	Immune suppression and angiogenesis	Multiple	Tumor growth
Immune exhaustion	PD-1/CTLA-4/TIM-3/LAG-3	T-cell dysfunction	Multiple	Disease progression

## Data Availability

No new data were generated or analyzed in this study. All discussed data are available in cited literature.
